# Molecularly Engineered Covalent Organic Frameworks for Hydrogen Peroxide Photosynthesis

**DOI:** 10.1002/anie.202200413

**Published:** 2022-02-25

**Authors:** Mingpu Kou, Yongye Wang, Yixue Xu, Liqun Ye, Yingping Huang, Baohua Jia, Hui Li, Jiaqi Ren, Yu Deng, Jiahao Chen, Ying Zhou, Kai Lei, Li Wang, Wei Liu, Hongwei Huang, Tianyi Ma

**Affiliations:** ^1^ College of Materials and Chemical Engineering Key Laboratory of Inorganic Nonmetallic Crystalline and Energy Conversion Materials China Three Gorges University Yichang 443002 China; ^2^ Hubei Three Gorges Laboratory 443007 Yichang China; ^3^ Engineering Research Center of Eco-environment in Three Gorges Reservoir Region Ministry of Education China Three Gorges University Yichang 443002 China; ^4^ Centre for Translational Atomaterials Swinburne University of Technology Hawthorn VIC 3122 Australia; ^5^ School of Science RMIT University Melbourne VIC 3000 Australia; ^6^ State Key Laboratory of Oil and Gas Reservoir Geology and Exploitation School of Oil & Natural Gas Engineering Southwest Petroleum University 610500 Chengdu China; ^7^ Key Laboratory of Material Chemistry for Energy Conversion and Storage (Ministry of Education) Hubei Key Laboratory of Material Chemistry and Service Failure Wuhan National Laboratory for Optoelectronics School of Chemistry and Chemical Engineering Huazhong University of Science and Technology (HUST) Luoyu Road Wuhan 430074 China; ^8^ Beijing Key Laboratory of Materials Utilization of Nonmetallic Minerals and Solid Wastes School of Materials Science and Technology China University of Geosciences Beijing 100083 P. R. China

**Keywords:** Bipyridine, COFs, Environmental Chemistry, H_2_O_2_, Photosynthesis

## Abstract

Synthesizing H_2_O_2_ from water and air via a photocatalytic approach is ideal for efficient production of this chemical at small‐scale. However, the poor activity and selectivity of the 2 e^−^ water oxidation reaction (WOR) greatly restricts the efficiency of photocatalytic H_2_O_2_ production. Herein we prepare a bipyridine‐based covalent organic framework photocatalyst (denoted as COF‐TfpBpy) for H_2_O_2_ production from water and air. The solar‐to‐chemical conversion (SCC) efficiency at 298 K and 333 K is 0.57 % and 1.08 %, respectively, which are higher than the current reported highest value. The resulting H_2_O_2_ solution is capable of degrading pollutants. A mechanistic study revealed that the excellent photocatalytic activity of COF‐TfpBpy is due to the protonation of bipyridine monomer, which promotes the rate‐determining reaction (2 e^−^ WOR) and then enhances Yeager‐type oxygen adsorption to accelerate 2 e^−^ one‐step oxygen reduction. This work demonstrates, for the first time, the COF‐catalyzed photosynthesis of H_2_O_2_ from water and air; and paves the way for wastewater treatment using photocatalytic H_2_O_2_ solution.

## Graphical abstract

A bipyridine‐based covalent organic framework photocatalyst (COF‐TfpBpy) showed excellent activity for H_2_O_2_ photosynthesis with a solar‐to‐chemical conversion efficiency of 0.57 % at 298 K and 1.08 % at 333 K. The photocatalytic process involves a 2 e^−^ water oxidation and 2 e^−^ oxygen reduction from water and air. The photocatalytic H_2_O_2_ solution can be directly used for Rhodamine B degradation and sterilization of *E. coli*.

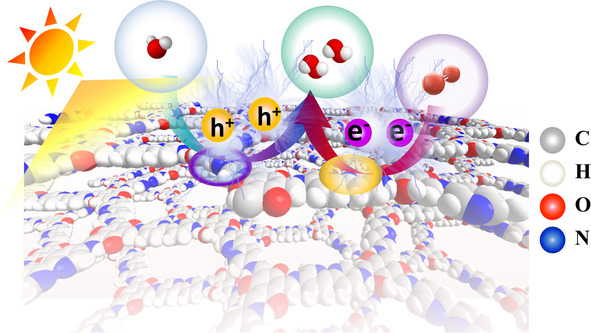

## Introduction

Hydrogen peroxide (H_2_O_2_) is widely used in the restoration of water environment as an environmentally friendly strong oxidant. It has been reported that many pollutants, including organic dyes, organochlorine pesticides, cyanides, phenols, antibiotics, microplastics, and personal care products, can be treated with H_2_O_2_.^[1]^ In addition, H_2_O_2_ can also be used as an environmentally friendly disinfectant to inactivate pathogenic microorganisms, since it does not cause secondary pollution to the environment.^[2]^ Due to the increased awareness in environmental protection and the current COVID‐19 pandemic, the demand for H_2_O_2_ is expected to increase substantially.

At present, the typical industrial production of H_2_O_2_ is based on anthraquinone method. However, this method involves expensive palladium‐based catalysts and complex reactions (catalyst hydrogenation and oxidation processes). It also requires a large amount of organic agents and generates toxic by‐products.^[3]^ Therefore, it is very important to develop the alternative approach for H_2_O_2_ manufacture based on efficient, economical and environmentally‐friendly process. In recent years, photocatalytic synthesis of H_2_O_2_ utilizing semiconductor catalysts has attracted significant interests.^[4]^ Visible‐light‐responsive non‐metal polymers have also been investigated as potential catalysts for H_2_O_2_ photosynthesis due to the low stability of H_2_O_2_ under UV light, heat, and in the presence of metal ions.^[5]^ Among the reported non‐metal photocatalysts,[^6^, ^7^] graphitic carbon nitride (g‐C_3_N_4_) is the most widely studied. This is attributed to the rich active sites of imine (C=N), which facilitate good catalytic activity for H_2_O_2_ photosynthesis.^[7]^ Without the presence of sacrificial reagents, however, the g‐C_3_N_4_ catalyzed photosynthetic production rate of H_2_O_2_ does not exceed 100 μM h^−1^ under one standard sun light. Although the catalytic performance of g‐C_3_N_4_ can be improved by composite, defect and single‐atom engineering,[^7^, ^8^] the efficiency of H_2_O_2_ production over g‐C_3_N_4_ based photocatalysts is still poor without the addition of sacrificial reagents or stabilizers.[^8b^, ^8c^, ^9^] Accordingly, the development of novel, efficient visible‐light‐responsive non‐metal semiconductor materials is of particular interest.

Covalent organic framework compounds (COFs) are a new type of visible‐light‐responsive non‐metallic polymer which have developed substantially in recent years.^[10]^ Preliminary studies have shown that COFs have great potential in photocatalytic hydrogen production and CO_2_ reduction,^[11]^ but there are few reports on its applications on the photocatalytic synthesis of H_2_O_2_.^[5d]^ Based on structural analysis, COFs have a highly ordered porous crystalline network structure to prevent the recombination of photogenerated electrons and holes, and the energy band and reactive sites can be structured at the molecular level. The structural characteristics impart these unique properties on COFs, which are known to improve catalyst activity. Therefore, COFs are envisaged as promising photocatalysts for the light‐driven synthesis of H_2_O_2_ without a sacrificial reagent.

In this work, bipyridine based COFs containing imine bonds are compared with g‐C_3_N_4_ (possessing a C=N reactive site) for the photocatalytic production of H_2_O_2_ from water and air. Firstly, we have verified that bipyridine based COFs featured a high activity for the production of H_2_O_2_ from water and air. The optimized photosynthetic rate of H_2_O_2_ reached 1042 μM h^−1^ under one standard sun light at 298 K, which was 496 times more efficient than that for pure g‐C_3_N_4_ (2.1 μM h^−1^). The apparent quantum yield (AQY) between 420–550 nm was greater than 8 %. The solar‐to‐chemical conversion (SCC) efficiency was 0.57 % for H_2_O_2_ synthesis, a value that substantially higher than the typical photosynthetic efficiency of plants (≈0.10 %).^[12]^ The SCC efficiency of the same catalyst at 333 K (1.08 %) was also higher than the highest value to date (1.0 %, no sacrifice reagents and buffer reagents).^[13]^ After 8 h of irradiation (the average duration of light in a day), the concentration of synthesized H_2_O_2_ reached 5.6 mM, which was directly used to degrade Rhodamine B (RhB) and inactivate *Escherichia coli* (*E. coli*). More importantly, we revealed the importance of bipyridine in COFs for H_2_O_2_ photocatalytic production from water and air. Bipyridine presents as active site, which is directly involved in the photocatalytic production of H_2_O_2_ via a 2 e^−^ one‐step redox reactions. Our findings revealed that COFs are excellent catalysts for the visible‐light‐driven production of H_2_O_2_, and this class of compounds may be a good starting point in the search for more active COFs photocatalysts.

## Results and Discussion

**Figure 1:**
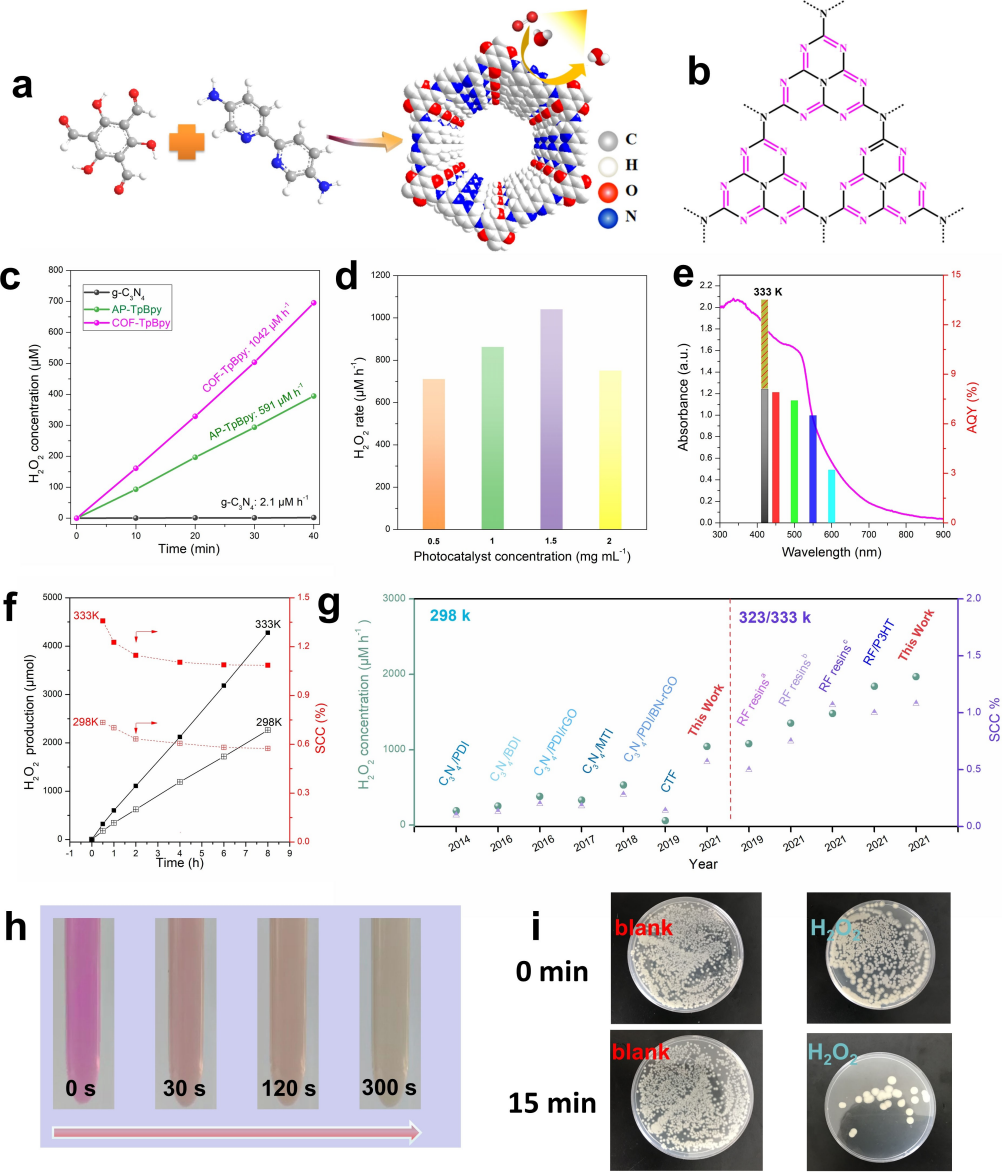
Photocatalytic performance of COF‐TfpBpy, AP‐TfpBpy and g‐C_3_N_4_ for H_2_O_2_ production from water and air. a) Schematic diagram of synthesis of COF‐TfpBpy with bipyridine active sites. b) Schematic structures of g‐C_3_N_4_ with C=N active sites. c) Photocatalytic activity of COF‐TfpBpy, AP‐TfpBpy and g‐C_3_N_4_ for H_2_O_2_ production in pure water. Conditions: *λ*>420 nm (298 K; xenon lamp, light intensity at 420–700 nm: 40.8 mW cm^−2^), water (10 ml), catalyst (15 mg). d) Photocatalytic activity of COF‐TfpBpy with different catalyst concentrations. Apparent quantum efficiency (e) and solar‐to‐chemical conversion efficiency (f) of COF‐TfpBpy. Conditions: *λ*>420 nm at 333 K or *λ*>300 nm at 298 K (xenon lamp, light intensity at 420–700 nm: 40.8 mW cm^−2^), water (400 ml), catalyst (600 mg). g) performance comparison of COF‐TfpBpy with other reported photocatalysts. h) RhB (10 mg L^−1^) decomposition in a photocatalytically produced H_2_O_2_ solution of via a fenton reaction. i) Sterilization of *E. coli* with a photocatalytically produced H_2_O_2_ solution.

Figure 1a and Figure 1b showed the molecular structures of bipyridine‐based COF and g‐C_3_N_4_, respectively. Bipyridine‐based COF (COF‐TfpBpy) and the corresponding amorphous polymer (AP‐TfpBpy) were prepared from 1,3,5‐triformylphloroglucinol (Tfp) and 2,2′‐bipyridine‐5,5′‐diamine (Bpy). Different from the XRD characteristic peak of AP‐TfpBpy, the strong XRD peak of COF at 2*θ*=3.68° corresponded to the (100) plane, indicating that the COF material has a better crystalline morphology (Figure S1a). Further characterization data of IR, ^13^C NMR and XPS confirmed the same chemical composition and bond structure of AP‐TfpBpy and COF‐TfpBpy, respectively (Figure S1b–d, Figure S2). SEM and TEM images showed that the crystals of COF‐TfpBpy and AP‐TfpBpy are both interlaced linear morphology, and COF‐TfpBpy has better crystallinity (Figure S3). And thus, it possesses a higher specific surface area than AP‐TfpBpy (Figure S4) and g‐C_3_N_4_ (Figure S5). X‐ray diffraction (XRD), infrared spectroscopy (IR), X‐ray photoelectron spectroscopy (XPS), scanning electron microscopy (SEM), transmission electron microscopy (TEM) indicated the successful preparation of typical g‐C_3_N_4_ samples (Figure S5–S7). For photocatalytic H_2_O_2_ production, water and air were employed as the oxygen and hydrogen sources. One xenon lamp (*λ*>420 nm; light intensity at 420–700 nm: 40.8 mW cm^−2^) was used as a light source. Peroxidase (POD) chromogenic method was used to determine the H_2_O_2_ concentrations due to the low interference with different electron sacrificial reagents addition, and the standard curve is drawn (Figure S8, S9).

Figure 1c revealed the photocatalytic activity of COF‐TfpBpy, AP‐TfpBpy and g‐C_3_N_4_. There is a clear linear relationship between H_2_O_2_ production and irradiation time. After 40 min of irradiation, the H_2_O_2_ concentration in the presence of COF‐TfpBpy reached 695 μM, which was about 1.8 and 496 times more efficient than that produced in the presence of AP‐TfpBpy (394 μM) and g‐C_3_N_4_ (1.4 μM), respectively. Figure 1d shows the photosynthetic rate of H_2_O_2_ in 10 ml water with different amounts of COF‐TfpBpy (5 mg, 10 mg, 15 mg, 20 mg) to assess the optimum catalyst concentration. It was found that 15 mg of COF‐TfpBpy (1.5 g L^−1^) furnished the highest photosynthetic rate of 1042 μM h^−1^, which is the best of those evaluated under one standard sun light (Table S1). The decreasing of H_2_O_2_ concentration alone with increasing concentrations of COF‐TfpBpy may be attributed to the fact that excess catalyst affects the light absorption of the reaction system. Figure 1e, f show the AQY and SCC efficiency of H_2_O_2_ production in water and air over COF‐TfpBpy, under the optimal catalyst concentration (1.5 g L^−1^ COF‐TfpBpy) in 400 mL water. At 298 K, the AQY@420 nm of COF‐TfpBpy was 8.1 %, with a total SCC efficiency of 0.57 %. At 333 K, the AQY@420 nm and SCC efficiency of COF‐TfpBpy were 13.6 % and 1.08 %, respectively, which were higher than the most efficient photocatalyst RF/P3HT (AQY, 10 %; SCC efficiency, 1.0 %; Figure 1g and Table S1).^[13]^ To the best of our knowledge, COF‐TfpBpy is the first COFs catalyst reported that can photocatalytically produce H_2_O_2_ without the presence of sacrificial reagents or stabilizers.^[5d]^

At present, the reported concentrations of H_2_O_2_ photocatalytically produced after 8 h (the average time of light in a day) are at the mM level, which can be used directly for environmental remediation.^[2]^ However, there were few reports on the use of H_2_O_2_ solutions generated by photocatalysis in treatment of pollutants, due to the presence of sacrificial reagents or buffers. In this work, after 8 h irradiation, the H_2_O_2_ concentration in COF‐TfpBpy system was 5.6 mM; and the H_2_O_2_ concentration was 5.4 mM even after five cycles (Figure S10a). The XRD and IR spectroscopy (Figure S10) on COF‐TfpBpy after the reaction showed that the catalyst had good stability in the photocatalytic process. When the equilibrium concentration of H_2_O_2_ reached 29.5 mM after 60 h irradiation (Figure S10b and c), the broadening of 26.5 degrees XRD peak (Figure S10d) indicated the slight exfoliation of the COF‐TfpBpy, although its photocatalytic activity was not affected. These findings indicated that COF‐TfpBpy is a recyclable photocatalyst. In order to verify the feasibility of the photocatalytically produced H_2_O_2_ solution for pollutant removal, RhB and *E. coli* were chosen as the target pollutants. Figure 1h shows that a 3.5 mL RhB solution (10 mg L^−1^) can be completely decomposed within 5 min by adding 0.5 mL (5.6 mM) of a photocatalytically produced H_2_O_2_ solution (Video S1). The growth of *E. coli* was effectively inhibited after 200 μL of a photocatalytically produced H_2_O_2_ solution was added into the plate **(**Figure 1i). These results revealed that the photocatalytic produced H_2_O_2_ solutions can be directly applied for the treatment of environmental pollutants with excellent performance.

**Figure 2:**
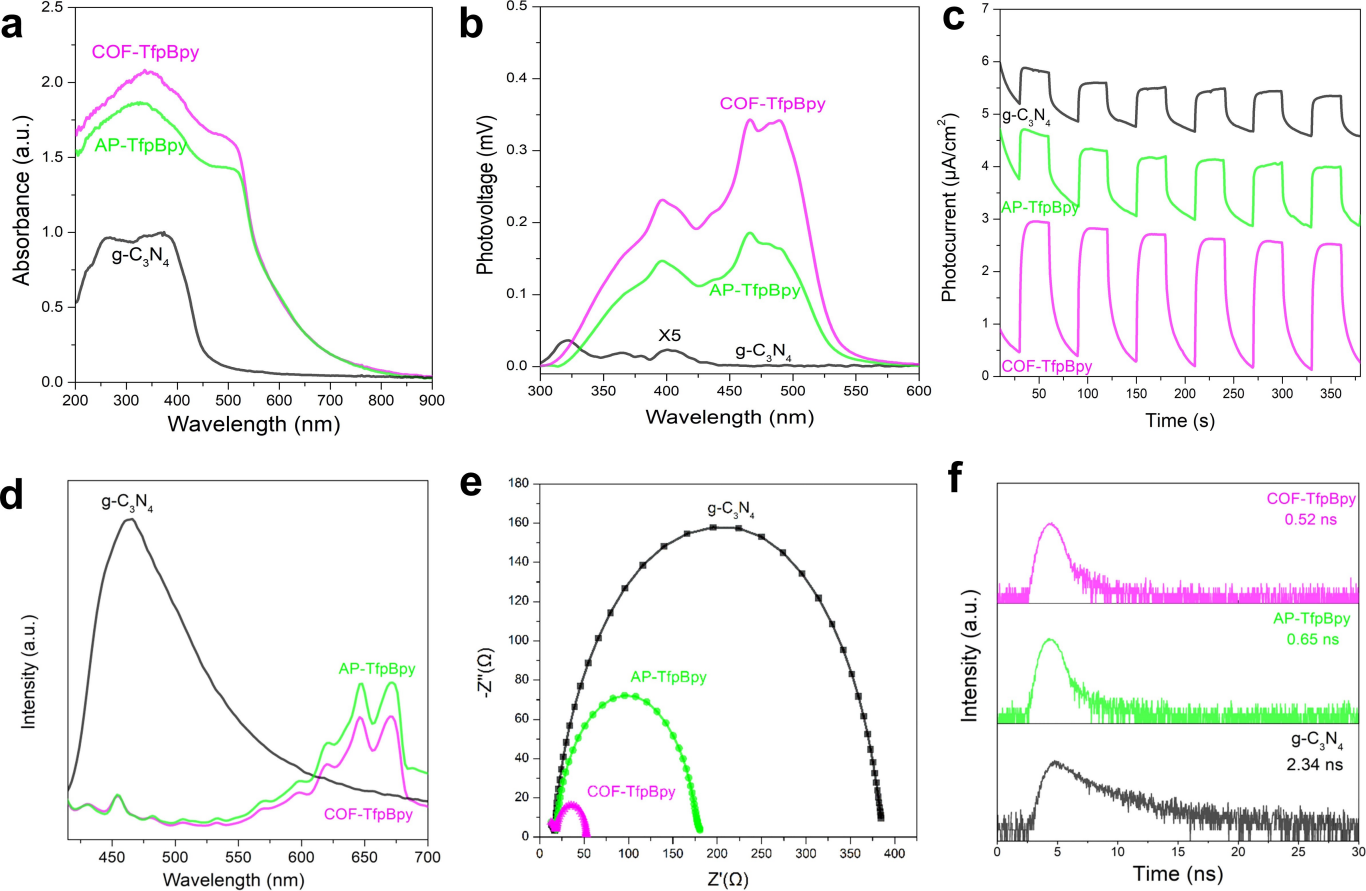
The efficiencies of light absorption and photogenerated carrier separation of COF‐TfpBpy, AP‐TfpBpy and g‐C_3_N_4_. a) DRS spectra. b) SPV spectra. c) Photocurrent. d) Fluorescence spectra. e) EIS spectra. f) TFS spectra.

The prerequisite for the occurrence of a catalytic reaction is that thermodynamics permits it. The band structure of COF‐TfpBpy, AP‐TfpBpy and g‐C_3_N_4_ were confirmed by experimental methods. Figure 2a displays the UV/Visible diffuse reflectance spectrum (DRS). It can be seen that COF‐TfpBpy and AP‐TfpBpy have a wider visible light absorption range than that of the g‐C_3_N_4_. In addition, surface photovoltage spectroscopy (SPV) revealed a high photoinduced voltage for COF‐TfpBpy (Figure 2b). According to the plots of (*a* *h* *v*)^1/2^ vs photon energy (*h* *v*), the band gap energies (*E*_g_) of COF‐TfpBpy, AP‐TfpBpy and g‐C_3_N_4_ were calculated as 2.37. 2.32 and 2.86 eV, respectively (Figure S11). According to the Mott–Schottky plots, the *E*_CB_ of COF‐TfpBpy, AP‐TfpBpy and g‐C_3_N_4_ were calculated as 0.21 V, 0.23 V and −0.67 V, respectively (Figure S12). Based on the equation *E*_g_=*E*_VB_−*E*_CB_, the VB positions of COF‐TfpBpy, AP‐TfpBpy and g‐C_3_N_4_ were calculated as 2.58, 2.55 and 2.20 V. As shown in Figure S13, the band structures of COF‐TfpBpy and AP‐TfpBpy were sufficient for the synthesis of H_2_O_2_ from H_2_O (*E* ${}_{H{}_{2}O{}_{2}/H{}_{2}O}$ =+1.78 V vs NHE) and O_2_ (*E* ${}_{O{}_{2}/H{}_{2}O{}_{2}}$ =+0.68 V vs NHE).^[12]^ On the other hand, COF‐TfpBpy also displayed higher wettability than that of the g‐C_3_N_4_ (Figure S14), which ensures the good dispersion in water for H_2_O_2_ photosynthesis.

Except for the high light absorption efficiency of COF‐TfpBpy and AP‐TfpBpy (Figure 2a and b), the efficiency of photogenerated carrier separation should be another important reason for the high activity of H_2_O_2_ photocatalysis.[^14^, ^15^] The photocurrent response is related to the separation rate of electrons and holes generated by light. As shown in Figure 2c, the photocurrent density of COF‐TfpBpy was significantly higher than that of AP‐TfpBpy and g‐C_3_N_4_, which indicated the COF‐TfpBpy had a higher charge separation efficiency and more usable surface carriers in photocatalytic reaction.^[15]^ Furthermore, the lower fluorescence intensity of COF‐TfpBpy indicated that the crystallization affects the separation efficiency of the photocarriers (Figure 2d).^[16]^ It also can been found that the multiple PL and SPV signals due to the wide molecular weight distribution and a large number of defects of COF‐TfpBpy and AP‐TfpBpy. Crystalline COF‐TfpBpy displays higher separation efficiency than amorphous AP‐TfpBpy and g‐C_3_N_4_. Electrochemical impedance spectroscopy (EIS) and transient fluorescence spectroscopy (TFS) are effective methods to evaluate the motion characteristics of carriers. As shown in Figure 2e and f, COF‐TfpBpy had a smaller charge transfer resistance, which lead to a faster interface electron transfer speed. In addition, the average relaxation lifetime of COF‐TfpBpy (0.52 ns) was lower than that of AP‐TfpBpy (0.65 ns) and g‐C_3_N_4_ (2.34 ns).^[14]^ It also indicated that photogenerated carriers of COF‐TfpBpy were more quickly captured by reactive substrates and thus were able to drive redox reactions, and the separation of electrons and holes in COF‐TfpBpy was particularly efficient.^[14]^ However, only a 1.8‐fold improvement in reaction rate (from 591 μM h^−1^ of AP‐TfpBpy to 1042 μM h^−1^ of COF‐TfpBpy) revealed that the crystallinity was not the key reason for the high performance of the bipyridine based polymers. On the contrary, the 281‐fold improvement in reaction rate, from 2.1 μM h^−1^ of g‐C_3_N_4_ to 591 μM h^−1^ of AP‐TfpBpy, indicated that the difference in active sites of photocatalysts was responsible for the high performance of the bipyridine based polymers.

**Figure 3:**
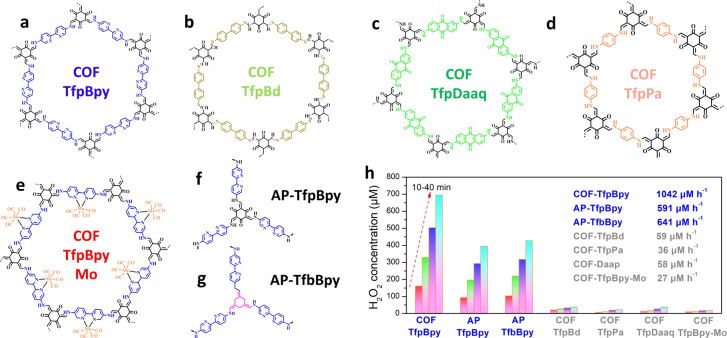
Chemical structures and photocatalytic performance of different COFs and APs for H_2_O_2_ photocatalytic production from water and air at 298 K. a) Structure of COF‐TfpBpy. b) Structure of COF‐TfpBd. c) Structure of COF‐TfpDaaq. d) Structure of COF‐TfpPa. e) Structure of COF‐TfpBpy‐Mo. f) Structure of AP‐TfpBpy. g) Structure of AP‐TfbBpy. h) H_2_O_2_ photocatalysis rates of different COFs and amorphous polymer photocatalysts.

In order to determine which monomer is the active site in COF‐TfpBpy **(**Figure 3a), the bipyridine monomer was replaced by benzidine (Bd), 2,6‐diaminoanthraquinone (Daaq) or p‐phenylenediamine (Pa) to form COF‐TfpBd (Figure 3b), COF‐TfpDaaq (Figure 3c) and COF‐TfpPa (Figure 3d), respectively. The structure, chemical bond, morphology, specific surface area and light absorption capacity of the prepared COF‐TfpBd, COF‐TfpDaaq and COF‐TfpPa were determined by PXRD, IR, ^13^C NMR, XPS, SEM, TEM and BET (Figure S15–S25), and the suitable band structures for photocatalytic H_2_O_2_ production by the above three COFs were determined by DRS and Mott–Schottky plots. Finally, the performance of these materials on H_2_O_2_ photocatalytic synthesis was studied. It was found that the activities were 59, 58 and 36 μM h^−1^ for COF‐TfpBd, COF‐TfpDaaq and COF‐TfpPa, respectively, all of which were far below the performance of COF‐TfpBpy and AP‐TfpBpy. On the other hand, Mo was used to block the nitrogen atom of 2,2′‐bipyridine of the COF‐TfpBpy (COF‐TfpBpy‐Mo) to evaluate the importance of the bipyridine monomer **(**Figure 3e and Figure S26–S29). The rate of H_2_O_2_ production in the presence of COF‐TfpBpy‐Mo was 26 μM h^−1^, which was also far below the performance of COF‐TfpBpy and AP‐TfpBpy **(**Figure 3f). It indicated that bipyridine is the active site of COF‐TfpBpy in the light driven reaction. Another 2,2′‐bipyridine based amorphous polymer (AP‐TfbBpy) formed from 1,3,5‐triformyl benzene (Tfb) and 2,2′‐bipyridine‐5,5′‐diamine (Bpy) was prepared to verify the importance of bipyridine active site (Figure 3g and Figure S30, S31). According to the XRD spectrum (Figure S30a), both AP‐TfpBpy and AP‐TfbBpy were amorphous, and the activity of AP‐TfbBpy was 641 μM h^−1^, which was very close to that of the AP‐TfpBpy (Figure 3h). It implied the importance of bipyridine in the photocatalysts for H_2_O_2_ production. In order to eliminate the influence of specific surface area on photocatalytic performances, the specific surface area rates of materials were compared. As shown in Figure S32, the photocatalytic H_2_O_2_ production of bipyridine based COFs (COF‐TfpBpy: 73.9 μM h^−1^ m^−2^; AP‐TfbBpy: 45.5 μM h^−1^ m^−2^) was much more efficient than that of the non‐bipyridine COFs (COF‐TfpDaaq: 5.9 μM h^−1^ m^−2^; COF‐TfpBd: 6.6 μM h^−1^ m^−2^; COF‐TfpPa: 3.9 μM h^−1^ m^−2^). And the linear bipyridine non‐porous polymer (surface area: 38.2 m^2^ g^−1^) also showed higher activity (247 μM h^−1^) than those of the g‐C_3_N_4_ and non‐bipyridine COFs (Figure S33). Therefore, the bipyridine active site, rather than the material porosity, determines the photocatalytic activity of COFs presented in this study.

**Figure 4:**
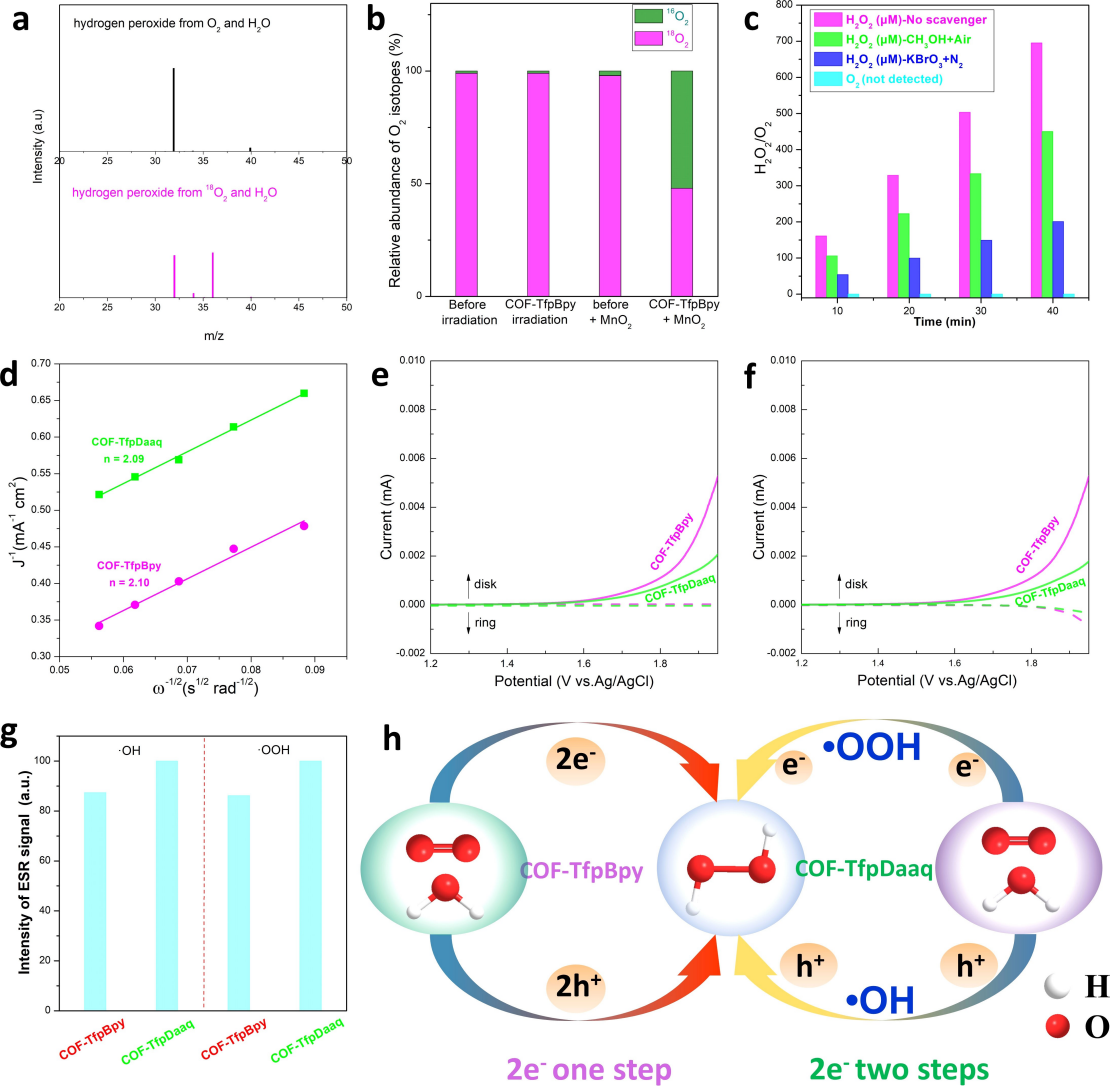
The effect of the bipyridine monomer on H_2_O_2_ photocatalysis. a) and b) ^18^O_2_ isotope experiment to explore the source of H_2_O_2_. c) Amount of O_2_ and H_2_O_2_ produced on COF‐TfpBpy in KBrO_4_ (0.1 M, as the electron acceptor) solution, and H_2_O_2_ produced on COF‐TfpBpy in CH_3_OH (10 % v/v, as the hole acceptor) solution. d) The Koutecky–Levich plots obtained by RDE measurements versus Ag/AgCl. e) and f) RRDE voltammograms obtained in 0.1 M phosphate buffer solution with a scan rate of 10 mV s^−1^ and a rotation rate of 1000 rpm. The potential of the Pt ring electrode is set at −0.23 V versus Ag/AgCl to detect O_2_. The potential of the Pt ring electrode is set to 0.6 V versus Ag/AgCl to detect H_2_O_2_. The oxidation current observed during RRDE tests indicates the oxidation of H_2_O_2_ occurs at the ring electrode. g) The intensity of ESR signal of ⋅OOH and ⋅OH by COF‐TfpBpy and COF‐TfpDaaq. h) The diagram of 2 e^−^ two‐steps and 2 e^−^ one‐step redox process.

To study the role of bipyridine in COFs for H_2_O_2_ photocatalytic production from water and air, the water oxidation and oxygen reduction were tested. Firstly, ^18^O_2_ isotope experiments are preformed (Figure 4a and b), no additional ^16^O_2_ was detected and almost no oxygen can be measured from WOR half reaction with 0.1 M KBrO_4_ as e^−^ trapping agent in Ar atmosphere (Figure 4c), which indicated that the 4 e^−^ oxidation of water to oxygen did not occur. On the other hand, the decomposition product of the reaction between photogenerated H_2_O_2_ and MnO_2_ consisted of 1 : 1 ^18^O and ^16^O. And the ORR half reaction rate (675 μM h^−1^) was about two folds treater than that of WOR rate (302 μM h^−1^). These indicated the atom utilization efficiency for the reaction between H_2_O and O_2_ (to generate H_2_O_2_) was close to 100 %, and the H_2_O_2_ photosynthesis underwent 2 e^−^ ORR and 2 e^−^ WOR pathways.

We further investigated the 2 e^−^ ORR and 2 e^−^ WOR pathways of COFs using rotating disk electrode (RDE) and rotating ring‐disk electrode (RRDE) measurements, respectively. As shown in Figure 4d and Figure S34, the average electron transfer number involved in oxygen reduction reactions were calculated to be 2.10 and 2.09 for COF‐TfpBpy and COF‐TfpDaaq, respectively. It indicated that 2 e^−^ ORR pathway was independent of bipyridine site. During RRDE tests, the potential of the rotating disk electrode was scanned from 1.2 to 1.95 V (vs Ag/AgCl) with a scan rate of 10 mV s^−1^, while a constant potential of −0.23 V was applied to the Pt ring electrode. In this setup, the O_2_ generated at the rotating disk electrode can be swept to the ring electrode where O_2_ can be reduced. As shown in Figure 4e, the increasing disk currents with potentials higher than 1.5 V (solid lines, vs Ag/AgCl) indicate that water oxidation occurred at the rotating disk electrode for both COF‐TfpBpy and COF‐TfpDaaq. No reduction currents were observed for COF‐TfpBpy and COF‐TfpDaaq at the Pt ring electrode, suggesting that COF‐TfpBpy and COF‐TfpDaaq cannot generate O_2_ via water oxidation (4 e^−^ WOR process). However, when the potential applied at the ring electrode was changed to an oxidative potential of +0.6 V, a much higher oxidation current can be observed for COF‐TfpBpy compared to COF‐TfpDaaq, due to H_2_O_2_ oxidation at the Pt ring electrode (Figure 4f). Hence, the RDE and RRDE measurements supported that H_2_O_2_ photosynthesis underwent 2 e^−^ ORR and 2 e^−^ WOR pathways for COFs.

(1) $$H{}_{2}O+h{}^{+}\to {}^{•}\mathrm{OH}+H{}^{+}$$

(2) $${}^{•}\mathrm{OH}+{}^{•}\mathrm{OH}\to H{}_{2}O{}_{2}$$

(3) $$2\;H{}_{2}O+2\;h{}^{+}\to H{}_{2}O{}_{2}+2\;H{}^{+}$$

(4) $$O{}_{2}+H{}^{+}+e{}^{-}\to {}^{•}\mathrm{OOH}$$

(5) $${}^{•}\mathrm{OOH}+e{}^{-}+H{}^{+}\to H{}_{2}O{}_{2}$$

(6) $$O{}_{2}+2\;e{}^{-}+2\;H{}^{+}\to H{}_{2}O{}_{2}$$

Whether COF has bipyridine site or not, it shows a 2 e^−^ redox process. But, bipyridine sites may result in different reaction steps for H_2_O_2_ photosynthesis. It has been reported two routes for ORR and two routes for WOR, which are possible for the photocatalytic synthesis of H_2_O_2_ from water and air via 2 e^−^ redox process. The WOR routes include a 2 e^−^ two‐steps process for H_2_O_2_ synthesis with ⋅OH as the intermediate species [Eq. (1) and (2)], and a 2 e^−^ one‐step WOR process for H_2_O_2_ generation [Eq. (3)]. ORR routes include a 2 e^−^ two‐steps ORR process for H_2_O_2_ synthesis with ⋅OOH as the intermediate species [Eq. (4) amd (5)], and a 2 e^−^ one‐step ORR process for H_2_O_2_ generation [Eq. (6)]. In order to study the effect of bipyridine site on the mechanisms of H_2_O_2_ production, ⋅OOH and ⋅OH from bipyridine based COF‐TfpBpy and non‐bipyridine based COF‐TfpDaaq were quantified. As shown in Figure 4g and Figure S35, 5,5‐dimethyl‐pyrroline N‐oxide (DMPO) was used as a free‐radical spin‐trapping agent in electron paramagnetic resonance (EPR) to measure ⋅OOH and ⋅OH. The ⋅OOH and ⋅OH signal intensity for both COF‐TfpBpy and COF‐TfpDaaq were comparable. However, compared with COF‐TfpDaaq, COF‐TfpBpy produced more H_2_O_2_ (Figure 3c). Therefore, it implied that H_2_O_2_ from bipyridine based COFs was mainly produced by a 2 e^−^ one‐step redox process; while the H_2_O_2_ was produced through a 2 e^−^ two‐steps process via ⋅OOH and ⋅OH intermediate species for non‐bipyridine based COFs. We further carried out a capture experiment of the active species to confirm the above arguments (Figure S36). The addition of benzoquinone (BQ, ⋅OOH scavenger) and tert‐butanol (⋅OH scavenger) did not result in significant effect on the amount of H_2_O_2_ generated in the presence of bipyridine based COF‐TfpBpy, AP‐TfbBpy and AP‐TfpBpy; yet the same reagents greatly inhibited the generation of H_2_O_2_ in the presence of non‐bipyridine based COF‐TfpDaaq, COF‐TfpBd and COF‐TfpPa. It thus proved the argument that bipyridine site changes the mode of H_2_O_2_ generation from a 2 e^−^ two‐steps process to a 2 e^−^ one‐step redox process. As those shown in Figure 4h, under light illumination of bipyridine based COF‐TfpBpy, H_2_O reacted directly with two holes to form H_2_O_2_, whilst O_2_ reacted directly with two electrons and two protons to form H_2_O_2_. The non‐bipyridine based COF‐TfpDaaq obtained H_2_O_2_ through intermediates (⋅OOH and ⋅OH) via a 2 e^−^ two‐steps redox process. Further investigations are still required to determine the exact mechanism of the 2 e^−^ one‐step redox process at the bipyridine site.

**Figure 5:**
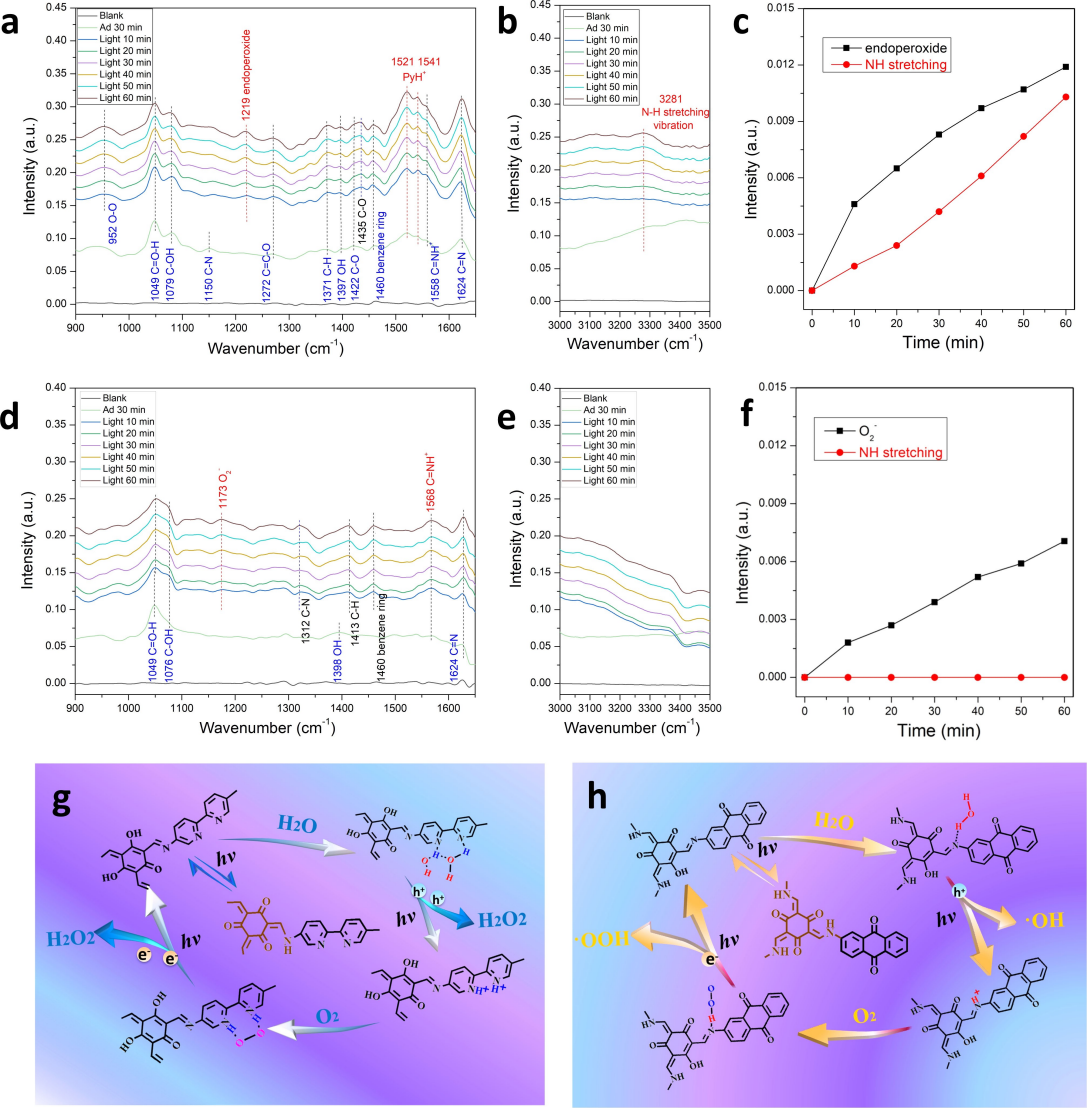
Photocatalytic mechanism of H_2_O_2_ synthesis in the presence of COF‐TfpBpy or COF‐TfpDaaq based on in situ FT‐IR. a) and b) In situ FT‐IR spectrum of COF‐TfpBpy for photosynthetic H_2_O_2_ production at 900–1650 cm^−1^ and 3000–3500 cm^−1^. c) The peak intensity of endoperoxide intermediate species and NH stretching vibration changes with increasing illumination time. d) and e) In situ FT‐IR spectrum of COF‐TfpBpy for photosynthetic H_2_O_2_ production at 900–1650 cm^−1^ and 3000–3500 cm^−1^. f) The peak intensity of O^2−^ and NH stretching vibrations changes with increasing illumination time. g) and h) Photocatalytic mechanism of H_2_O_2_ synthesis in the presence of COF‐TfpBpy and COF‐TfpDaaq.

In situ Fourier transform infrared (in situ IR) spectrometry is an effective method to understand the photocatalytic mechanism. Figure 5a–f show the in situ IR spectrometry of COF‐TfpBpy and COF‐TfpDaaq for H_2_O_2_ photocatalysis under a continuous steam‐saturated O_2_ flow. After the system was equilibrated for 30 min, vibrations corresponding to C−OH (1076 cm^−1^), O−H (1397 cm^−1^), C−O (1422 cm^−1^), and C=N (1624 cm^−1^) for COF‐TfpBpy were apparent. For COF‐TfpDaaq, vibrations corresponding to C−OH (1076 cm^−1^), O−H (1398 cm^−1^), and C=N (1624 cm^−1^) were also apparent.[^16^, ^17^] However, the intensity of these signals for COF‐TfpDaaq were very low. In contrast, the higher signal intensities indicated the conversion of COF‐TfpBpy from the keto‐amine form into the enol‐imine form under aqueous conditions.^[17]^ The structural changes of COF‐TfpBpy also gave rise to the signals corresponding to C=O−H (1049 cm^−1^), C−N (1150, 1292 cm^−1^), C=C−O (1272 cm^−1^), C−H (1371 cm^−1^) and the benzene ring (1460 cm^−1^). More importantly, there were strong vibrations for PyH^+^ (1521, 1541 cm^−1^) and C=NH^+^ (1558 cm^−1^) in COF‐TfpBpy,[^16^, ^18^] that were not observed from the spectra of COF‐TfpDaaq. These implied that bipyridine is an important monomer for enol‐to‐keto tautomerism due to the strongly adsorbed H_2_O molecules on the bipyridine nitrogen of COF‐TfpBpy. On the contrary, Daaq and reported phenyl monomers were unable to result in the structural change from keto‐amine form into enol‐imine form of imine‐based COFs.^[19]^ This may be the origin of H_2_O_2_ photocatalysis over COF‐TfpBpy, because water oxidation is the rate‐determining step.

Under light irradiation, the signal intensity corresponding to C=N, C−OH and C=NH^+^ increased, and particularly pronounced signals are also apparent for COF‐TfpDaaq (Figure S37). It indicated that light also induced the transformation of the keto‐amine form into the enol‐imine form.[^17^, ^20^] Meanwhile, some structural transformation induced infrared vibration have shifted (such as 1422 to 1435 cm^−1^ for C−O) for COF‐TfpBpy, and new infrared vibration appeared (C−N: 1312 cm^−1^; C−O: 1413 cm^−1^; benzene ring: 1460 cm^−1^) for COF‐TfpDaaq due to the structural transformation accelerating. However, these apparent structural transformations did not affect the H_2_O adsorption on the bipyridine nitrogen. New infrared vibration signals at 952, 1219 and 3281 cm^−1^ for COF‐TfpBpy can be attributed to the O−O bonding, endoperoxide intermediate species and the NH stretching vibration, respectively.^[21]^ The intensity of these new infrared vibrations gradually increased along with the increase in illumination time. Combined with the increased vibrations of PyH^+^ (1521, 1541 cm^−1^) and the appeared (BPy)(H_2_O)_*n*_ clusters (Figure S38),^[22]^ the 2 e^−^ one‐step redox process over COF‐TfpBpy can be summarized as Figure 5g. Initially, the COF‐TfpBpy undergoes light‐induced structural transformation. Two bipyridine nitrogen atoms adsorb two water molecules, and hydrogen bonds are formed between the two water molecules. Photoexcitation leads to the oxidation of the two water molecules to generate H_2_O_2_, leaving two protonated pyridine substituents (PyH^+^).^[23]^ An oxygen molecule is then adsorbed on the PyH^+^ substituent of COF‐TfpBpy to form an endoperoxide intermediate (N−H−O−O−H−N), which is easily transformed into H_2_O_2_ via a selective 2 e^−^ reduction. The enhanced activity of protonated COF‐TfpBpy and its absence of activity in nonaqueous solvents for H_2_O_2_ production also support that the initial step is a 2 e^−^ water oxidation (Figure S39). Compared with COF‐TfpBpy, new peaks for COF‐TfpDaaq were apparent at 1173 cm^−1^ and 1568 cm^−1^, which corresponded to O^2−^ and C=NH^+^, respectively.[^20^, ^24^] O^2−^ is the single electron reduction product from O_2_, which indicates that H_2_O_2_ was photocatalytically produced by a 2 e^−^ two‐steps process with COF‐TfpDaaq. As shown in Figure 5h, the structure of COF‐TfpDaaq also undergoes light‐induced transformation. Then, the nitrogen atom of C=N adsorbs a single water molecule. After photoexcitation, the water molecules are oxidized to generate ⋅OH and protonate the imine (C=NH^+^). Two ⋅OH radicals can couple to generate one H_2_O_2_. A single oxygen molecule can be adsorbed on the C=NH^+^ of COF‐TfpDaaq to form the O^2−^ intermediate species, and O^2−^ is transformed into ⋅OOH via a single‐electron reduction. Then, the ⋅OOH radical is converted to H_2_O_2_ via another one‐electron reduction. The non‐dominant 2 e^−^ two‐steps process of COF‐TfpBpy catalyst is not considered due to the high selectivity of the 2 e^−^ route for photocatalytic H_2_O_2_ production.

In order to account for the difference in selectivity, DFT calculation was used to compare the adsorption energies of H_2_O and O_2_ on different N sites of COF‐TfpBpy. As shown in Figure S40, O_2_ cannot adsorb onto the N sites of COF‐TfpBpy without light irradiation. However, the adsorption energy (−0.677 eV) for two H_2_O molecules onto bipyridine nitrogen atoms was lower than that of one H_2_O molecule on an imine nitrogen atom (−0.384 eV) or one H_2_O molecule on a bipyridine nitrogen atom (−0.376 eV). It indicated that the bipyridine site was favorable for simultaneous adsorption of two water molecules to accelerate the 2 e^−^ one‐step WOR under light irradiation. After protonation of the catalyst to C=NH^+^ or PyH^+^, O_2_ can be adsorbed on H sites of C=NH^+^ or PyH^+^. The adsorption energy (−2.702 eV) of molecular O_2_ on bipyridine via the endoperoxide intermediate species was much lower than that of molecular O_2_ on bipyridine (−1.243 eV) or C=NH^+^ via the O^2−^ intermediate species (−0.376 eV). It indicates that bipyridine protonation was favorable for O_2_ adsorption via endoperoxide intermediate species and accelerated the oxygen reduction reaction under light irradiation. The presence of bipyridine in the photocatalyst induced the high selectivity of the 2 e^−^ one‐step route for H_2_O_2_ production.

## Conclusion

In summary, we reported the crystalline polymer photocatalyst (COF‐TfpBpy) for the efficient photocatalytic production of H_2_O_2_ without sacrificial reagents and stabilizers. The photocatalytic H_2_O_2_ solution can be directly used in the pollutant removal and water disinfection. Our investigations revealed that protonation of the nitrogen atom in the bipyridine monomer facilitated the formation of suitable intermediates for H_2_O_2_ formation via 2 e^−^ one‐step redox reactions. The resulting bipyridine‐based polymer photocatalyst exhibited excellent activity for H_2_O_2_ production with 100 % atom utilization efficiency. Our findings provide important insights into the design and synthesis of bipyridine‐based polymer photocatalysts at the molecular level, and could be an excellent starting point to develop superior H_2_O_2_ photocatalysts.

## Conflict of interest

The authors declare no conflict of interest.

1

## Supporting information

As a service to our authors and readers, this journal provides supporting information supplied by the authors. Such materials are peer reviewed and may be re‐organized for online delivery, but are not copy‐edited or typeset. Technical support issues arising from supporting information (other than missing files) should be addressed to the authors.

Supporting Information

Supporting Information

## Data Availability

The data that support the findings of this study are available from the corresponding author upon reasonable request.
