# Supplementary material for: Molecularly Engineered Covalent Organic Frameworks for Hydrogen Peroxide Photosynthesis
Source: Angew Chem Int Ed Engl. 2022 Feb 25;61(19):e202200413. doi: 10.1002/anie.202200413 (PMC9305556; doi:10.1002/anie.202200413)
Supplement: Supplementary file 1 — Supporting Information [file ANIE-61-0-s002.pdf]

Angewandte Chemie
Eine Zeitschrift der Gesellschaft Deutscher Chemiker

Supporting Information

Molecularly Engineered Covalent Organic Frameworks for Hydrogen Peroxide Photosynthesis

M. Kou, Y. Wang, Y. Xu, L. Ye, Y. Huang, B. Jia, H. Li, J. Ren, Y. Deng, J. Chen, Y. Zhou, K. Lei, L. Wang, W. Liu, H. Huang, T. Ma**

Author Contributions

M.K. Data curation:Lead; Formal analysis:Lead; Investigation:Lead; Software:Lead; Writing – original draft:Lead
Y.W. Data curation:Equal; Investigation:Equal; Writing – original draft:Equal
Y.X. Data curation:Equal; Investigation:Equal; Methodology:Equal; Writing – original draft:Equal
L.Y. Funding acquisition:Lead; Resources:Supporting; Supervision:Lead; Writing – review & editing:Lead
Y.H. Supervision:Supporting; Writing – review & editing:Supporting
B.J. Funding acquisition:Supporting; Supervision:Supporting; Writing – review & editing:Supporting
H.L. Methodology:Supporting; Software:Supporting; Writing – review & editing:Supporting
J.R. Data curation:Supporting; Formal analysis:Supporting
Y.D. Software:Supporting; Validation:Supporting
J.C. Investigation:Supporting; Methodology:Supporting
Y.Z. Resources:Supporting; Supervision:Supporting
K.L. Resources:Supporting; Writing – review & editing:Supporting
L.W. Resources:Supporting; Writing – review & editing:Supporting
W.L. Resources:Supporting; Visualization:Supporting; Writing – review & editing:Supporting
H.H. Supervision:Supporting; Visualization:Supporting; Writing – review & editing:Supporting
T.M. Funding acquisition:Lead; Supervision:Lead; Writing – review & editing:Lead

Supporting Information for

Molecularly engineered covalent organic frameworks for hydrogen peroxide photosynthesis

Mingpu Kou[1#], Yongye Wang[1,#], Yixue Xu[1,2#], Liqun Ye[1,2,*], Yingping Huang[3], Baohua Jia[4,5], Hui Li[4,5], Jiaqi Ren[1], Yu Deng[1], Jiahao Chen,[6] Ying Zhou[6], Kai Lei[7], Li Wang[1], Wei Liu[1,2], Hongwei Huang[8], Tianyi Ma[4,5*]

1 College of Materials and Chemical Engineering, Key Laboratory of Inorganic Nonmetallic Crystalline and Energy Conversion Materials, China Three Gorges University, 443002 Yichang, China.

2 Hubei Three Gorges Laboratory, 443007 Yichang, China.

3 Engineering Research Center of Eco-environment in Three Gorges Reservoir Region, Ministry of Education, China Three Gorges University, Yichang 443002, China.

4 Centre for Translational Atomaterials, Swinburne University of Technology, Hawthorn, VIC 3122, Australia

5 School of Science, RMIT University, Melbourne, VIC 3000, Australia

6 State Key Laboratory of Oil and Gas Reservoir Geology and Exploitation, School of Oil & Natural Gas Engineering, Southwest Petroleum University, 610500 Chengdu, China.

7 Key Laboratory of Material Chemistry for Energy Conversion and Storage (Ministry of Education), Hubei Key Laboratory of Material Chemistry and Service Failure, Wuhan National Laboratory for Optoelectronics, School of Chemistry and Chemical Engineering, Huazhong University of Science and Technology (HUST), Luoyu Road, Wuhan, 430074, China.

8 Beijing Key Laboratory of Materials Utilization of Nonmetallic Minerals and Solid Wastes, School of Materials Science and Technology, China University of Geosciences, Beijing, 100083 P. R. China

[#] These authors contributed to the work equally.

*Correspondence author: E-mail: lqye@ctgu.edu.cn; tianyi.ma@rmit.edu.au

Contents

Part 1 Experimental Section

Part 2 Characterizations of COF-TfpBpy and AP-TfpBpy

Part 3 Characterizations of g-C_3N_4

Part 4 Standard curve of H_2O_2

Part 5 Catalytic activity and stability

Part 6 AQY and SCC

Part 7 Band structure of g-C_3N_4, COF-TfpBpy and AP-TfpBpy

Part 8 Wettability of the COF-TfpBpy

Part 9 Characterizations of COF-TfpDaaq

Part 10 Characterizations of COF-TfpBd

Part 11 Characterizations of COF-TfpPa

Part 12 Characterizations of COF-TfpBpy-Mo

Part 13 Characterizations of AP-TfbBpy

Part 14 The influence of specific surface area and porosity

Part 15 Average electron transfer number of ORR

Part 16 ESR and Capture experiment of active species

Part 17 In-situ Fourier transform infrared (in situ IR)

Part 18 The importance of protonation for H_2O_2 photosynthesis

Part 19 Theoretical calculation

Part 20 References

Part 1 Experimental Section

Materials. 2,2′-bipyridine-5,5′-diamine (Bpy, >97%) and 1,3,5-triformylphloroglucinol (Tp, >97%) were from Shanghai Tensus Biotech Co., Ltd. N, N-dimethylacetamide (DMAC, 99.8%, Extra Day, with molecular sieves, water ≤50 ppm) was provided by Energy Chemical. o-dichlorobenzene (o-DCB, 99%), acetic acid (AcOH, ≥99.8%) and 2,6-Diaminoanthraquinone (Daaq) were purchased from Aladdin.

Synthesis of COF-TfpBpy. 1,3,5-Triformylphloroglucinol (Tfp) and 2, 2'-bipyridine-5, 5'-diamine (Bpy) were put into a 10 mL glass ampoule. Then a mixed solvent of N,N-dimethylacetamide (DMAC) and dichlorobenzene (3:1) were also added, and ultrasonically make it uniformly dispersed. Then quickly added 0.6 mL of 6 M acetic acid (AcOH) aqueous solution and sonicate again. After degassing, the reaction was conducted at 120 °C for 3 days. The solid was then washed away with a large amount of DMAC, acetone and dichloromethane and dried under vacuum.[1]

Protonation of COF-TfpBpy. 20mg COF-TfpBpy was dispersed in 2 mL 1M HCl, stirred vigorously for 12 h, washed with pure water to neutrality, and freeze-dried for 12 h.

Synthesis of AP-TfpBpy. 1,3,5-Triformylphloroglucinol (Tfp) and 2, 2'-bipyridine-5, 5'-diamine (BPY) were put into a 10 mL glass ampoule. Then added dioxane solvent and sonicate to make it evenly dispersed. Then quickly added 0.6 mL of 6 M acetic acid (AcOH) aqueous solution and sonicate again. After degassing, the reaction was conducted at 120 °C for 3 days. The solid was then washed away with a large amount of DMAC, acetone and dichloromethane and dried under vacuum.

Synthesis of COF-TfpDaaq. 1,3,5-triformylphloroglucinol (Tfp) and 2,6-Diaminoanthraquinone (Daaq) were put into a 10 mL glass ampoule. Then a mixed solvent of N,N-dimethylacetamide (DMAC) and mesitylene (3:1) were also added, and ultrasonically make it uniformly dispersed. Then quickly added 0.05 mL of 6 M aqueous acetic acid (AcOH) and sonicate again. After degassing, the reaction was conducted at 120 °C for 3 days. The catalyst was cleaned several times with 1, 4-dioxane, tetrahydrofuran and acetone, and dried under vacuum at 80 °C for 12 h.[2]

Synthesis of COF-TfpBd. 1,3,5-triformylphloroglucinol (Tfp) and Benzidine (BD) were put into a 10 mL glass ampoule. Then add a mixed solvent of mesitylene and dioxane (1:1), and ultrasonic treatment to make it evenly dispersed. Then quickly add

0.5 mL of 3 M acetic acid (AcOH) in water and sonicate again. After degassing, react at 120°C for 3 days. Then a large amount of acetone was used to wash off the solid and dried under vacuum.[3]

Synthesis of COF-TfpBpy-Mo. COF-TfpBpy (20 mg) and $Mo(CO)_6$ (11 mg) were dispersed in 20 ml toluene solution and reacted at 110 °C for 6 h under the protection of N_2 atmosphere. After cooling to room temperature, it was washed with methanol and water, and vacuum dried at 80 °C for 12 h.

Synthesis of AP-TfbBpy. 1,3,5-Triformylbenzene (Tfb) and 2,2'-bipyridine-5,5'-diamine (BPY) were put into a 10 mL glass ampoule. Then added dioxane solvent and sonicate to make it evenly dispersed. Then quickly added 0.6 mL of 6 M acetic acid (AcOH) aqueous solution and sonicate again. After degassing, reacting at 120°C for 3 days. The solid was then washed away with a large amount of DMAC, acetone and dichloromethane and dried under vacuum.

Synthesis of COF-TfpPa. 1,3,5-triformylphloroglucinol (Tfp) and p-phenylenediamine (Pa) were put into a 10 mL glass ampoule. Then added a mixed solvent of mesitylene and dioxane (1:1), and ultrasonic treatment to make it evenly dispersed. Then quickly added 0.25 mL of 6 M acetic acid (AcOH) in water and sonicate again. After degassing, reacting at 120 °C for 3 days. Then a large amount of acetone was used to wash off the solid and dried under vacuum.[4]

Synthesis of linear bipyridine non-porous polymer. In a single neck round-bottom flask, 16.2 mg (0.15 mmoL) of p-phenylenedialdehyde, 31.8 mg (0.15mmoL) of 2, 2'-bipyridine-5, 5'-diamine (Bpy) was added in 20 ml of anhydrous ethanol. 3ml of glacial acetic acid was subsequently added to the above mixture as the catalyst. Then the mixture was heated to 75 °C and was stirred magnetically. The reaction lasted for 4 h, after which the mixture was cooled down to room temperature. Finally, the insoluble matter is filtered out and washed with a large amount of DMF followed by washing with water, ethanol and acetone. The filtered solid powder was vacuum dried at 70 °C for 12 h.

Characterization. The morphologies and microstructures of Catalytic materials were characterized by JEOL JEM-2100F (URH) Field Emission Electron Microscope (TEM) and JSM-7500F Emission Electron Microscope (SEM). XRD patterns were obtained by a Bruker AXS D8 Advance powder diffractometer using Ni filtered Cu Kα radiation (3 °/min). The BEL SORP-MAX volume adsorption analyzer and

analyzer (Novatouch, Quantachrome) were used to carry out N_2 (77K) adsorption-desorption measurement and BET analysis. X-ray photoelectron spectrum (XPS) of samples (A1Ka, 150 W, C1s 284.6 eV) were determined by X-ray photoelectron spectrometer (Thermos Scientific ESCALAB 250XI). The UV-Vis absorption spectra were recorded using UH4150 (HITACHI) was used to determine the DRS in the range of 200-800 nm. Time-resolved PL spectra was recorded by a FLS980 multifunction steady state and transient state fluorescence spectrometer. The electrochemical impedance and photocurrent Response of the catalysts were measured on electrochemical workstation (CHI660D, CHI Instruments, Shanghai, China). The Fourier transform infrared (FTIR) spectrum of the samples was acquired from a Nicolet iS-50 instrument at the scan range of 400-4000 cm^{-1}. The solid phase 13C NMR spectra were obtained on an Agilent 600 M solid state NMR spectrometer.

H_2O_2 photocatalysis. 5 mg catalyst and 10 ml water were evenly dispersed in a quartz bottle with a volume of 50 ml and passed into dry air, stirred for 30 min under dark conditions to reach adsorption-desorption equilibrium, and then irradiated with a xenon lamp source (PLS-SXE300D, Beijing Perfectlight). After sampling every 10 minutes, filter with a 0.22 μm filter to detect the content of H_2O_2 in the solution.

H_2O_2 detection methods. A phosphate buffer was prepared by dissolving 2.8756 g $K_2HPO_4·3H_2O$ and 11.935 g KH_2PO_4 in 200 ml deionized water. Then prepared N, N-diethyl-1,4-phenylenediamine sulfate (DPD, 97%, Aldrich) and peroxidase (POD, horseradish, Aldrich) stock solutions. (Dissolve 0.1 g DPD in 10 ml 0.05 M H_2SO_4 solution and 10 mg POD in 10 ml deionized water.) During the experiment, take 2.5ml sample solution into a quartz tube each time, added 0.4 ml phosphate buffer and 50 μL POD solution in sequence. Liter and 50 microliters of DPD solution, mix well. Useing UV-2600 (Shanghai Tianmei Scientific Instrument Co., Ltd.) to measure absorbance at 552 nm. Calibrate the H_2O_2 concentration by diluting a 30% H_2O_2 stock solution.[5]

RhB degradation. 10 mg Rhodamine B (RhB) and 278 mg $FeSO_4·7H_2O$ were dissolved in a 1 L volumetric flask. Taking out 3.5 ml RhB solution and put it in a 10 ml centrifuge tube, and then 0.5 ml photocatalytic H_2O_2 solution was gradually added. The color change of RhB was recorded by video.

Sterilization of *E. coli*. Escherichia coli (*E. coli*) was cultured in LB broth for 16 h in a shaking incubator. Then, the bacterial liquid was centrifuged (10,000 rpm, 1 min) with 0.85% physiological saline and washed twice. 3 mL Bacterial liquid, 0.5 ml

photocatalytic H_2O_2 solution and 0.1 mL $FeSO_4 \cdot 7H_2O$ (0.1 mM) were added a 10 ml centrifuge tube, the concentration of the bacterial in the system was about 10^5 cfu/ mL. Took 100 μL bacterial solution every 15 min. The obtained samples were diluted to an appropriate concentration, and then 100 μL of bacterial solution was applied to the agar plate. At least, the agar plates were incubated for 12 h at 37 °C in a biochemical tank.

Photoelectrochemical measurements: The Mott–Schottky plots, photocurrent response and electrochemical impedance of the catalysts were measured on an electrochemical workstation (CHI660E, CHI Instruments, Shanghai, China). A 300 W Xe lamp was utilized as the light source and Na_2SO_4 (0.5 M, pH=6.5) aqueous solution was used as the supporting electrolyte throughout the photocurrent measurements. A platinum wire and Ag/AgCl electrode were used as, counter electrode and reference electrode, respectively. 0.003 g ethyl cellulose was dissolved in about 10 mL ethanol with 0.03 g catalyst. Then, a glass stick was applied to FTO with a layer of high-temperature adhesive tape on the edge, followed by drying in air and activation at 120 °C for 3 h.

$$E\text{（}VS.\ RHE\text{）}=E\text{（}VS.\ Ag/AgCl\text{）}+0.197\ V+0.0591*pH$$

In-situ Fourier transform infrared spectrometer (FT-IR) analysis: In-situ Fourier transform infrared spectrometry was performed on a Nicolet iS-50 instrument. The sample was filled into an in-situ IR cell, and O_2 and H_2O vapors were introduced into the cell and fiber source (FX300, Beijing Perfect Light Technology Co., Ltd., Beijing, China) through the CaF_2 window of the cell. Before the measurement, the samples were degassed at 423 K for 4 h. The baseline was obtained before the sample reached O_2 adsorption equilibrium within 1 h.

Mass spectrometry method: The $^{18}O_2$ isotope was measured on the mass spectrometer (Shimadzu GCMS-QP2010 Plus). Adding 5 ml of $^{18}O_2$, 3 ml of H_2O, and 7.5 mg of catalyst COF-TfpBpy to the reactor (5 ml) connected to the mass spectrometer, and the mixture was evenly mixed and irradiated with a 300 w xenon lamp (λ > 420 nm) for 5 h. After injecting N_2 to rule out O_2 in a new reactor (5 ml) with 2 mL above photocatalytic H_2O_2 solution, an appropriate amount of MnO_2 was adding to generate O_2. At end, the O_2 was detected by mass spectrometry.

Computational details: The calculations were carried out using density functional theory (DFT) implemented via the Vienna Ab-initio Simulation Package (VASP). The

generalized gradient approximation (GGA) functional and Perdew−Burke−Ernzerhof (PBE) functional were used for the electronic exchange and correlation effects. Modifing Gaussian path and smearing width is 0.2 eV.

Geometries were optimized until the energy and the force were converged to 1.0×10^{-5} eV/atom and 0.05 eV/Å, respectively. An energy cutoff was set as 400 eV for the plane-wave expansion of the electronic wave function. A vacuum region of 15 Å was introduced to avoid the interactions between the periodic slabs. Besides, k-point grid is set to 3 * 2 * 1.

The adsorption energy (E_{ads}) of O_2 or H_2O molecule on the surface is calculated as follow:

$$E_{ads}=E_{total}-E_{surface}-E_{O_2}\ (E_{H_2O})$$

where E_{total} represents the energy of surface with adsorbed O_2 or H_2O molecule, $E_{surface}$ and E_{O_2} or E_{H_2O} represent the energies of isolated surface and O_2 or H_2O, respectively.

Part 2 Characterizations of COF-TfpBpy and AP-TfpBpy

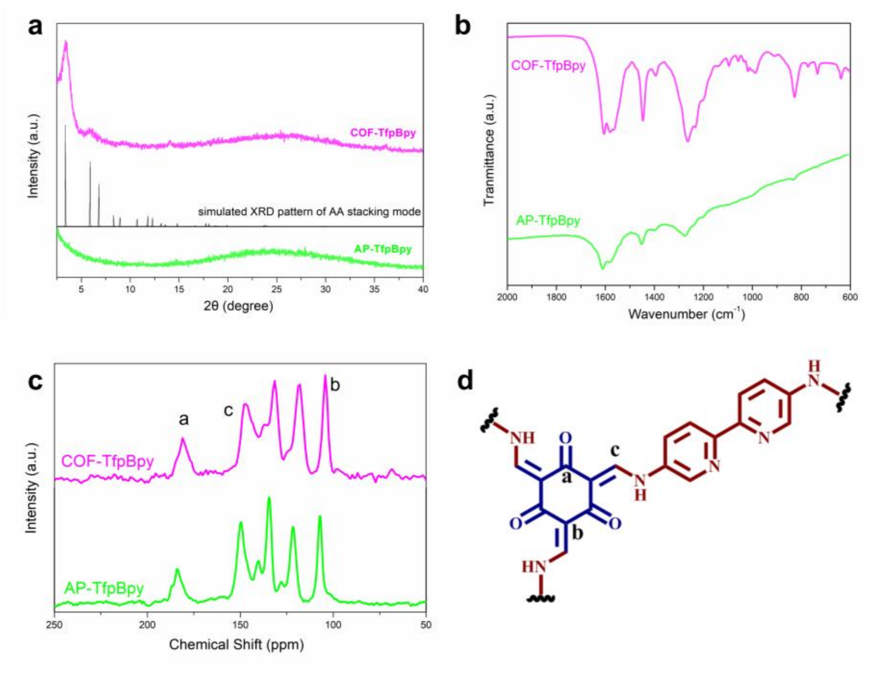

Figure S1 The XRD (a), IR (b) and solid state ^{13}C NMR (c, d) spectra of COF-TfpBpy and AP-TfpBpy.

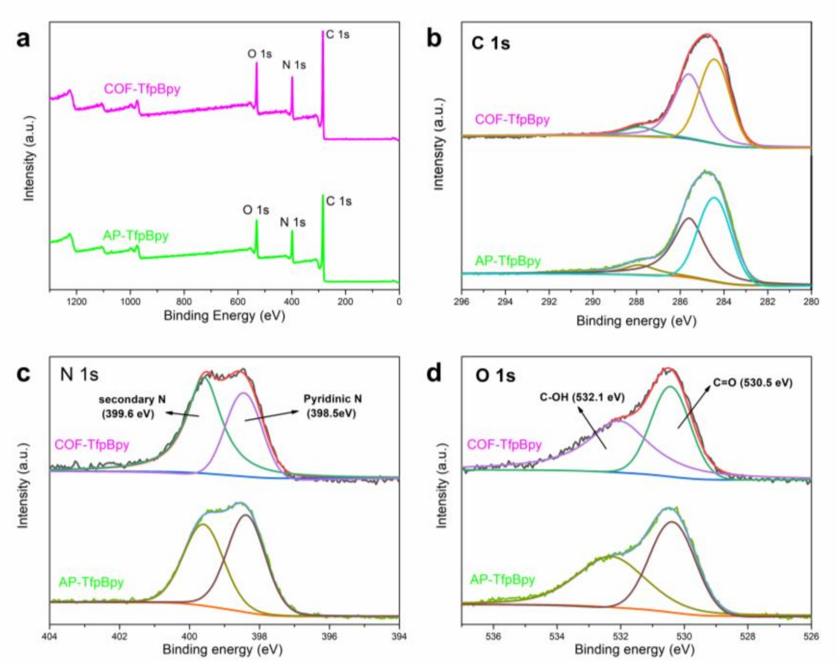

Figure S2 The XPS spectrum of COF-TfpBpy and AP-TfpBpy. Survey scan XPS profile (a), C 1s (b), N 1s (c) and O 1s (d) XPS spectrum of COF-TfpBpy and AP-TfpBpy.

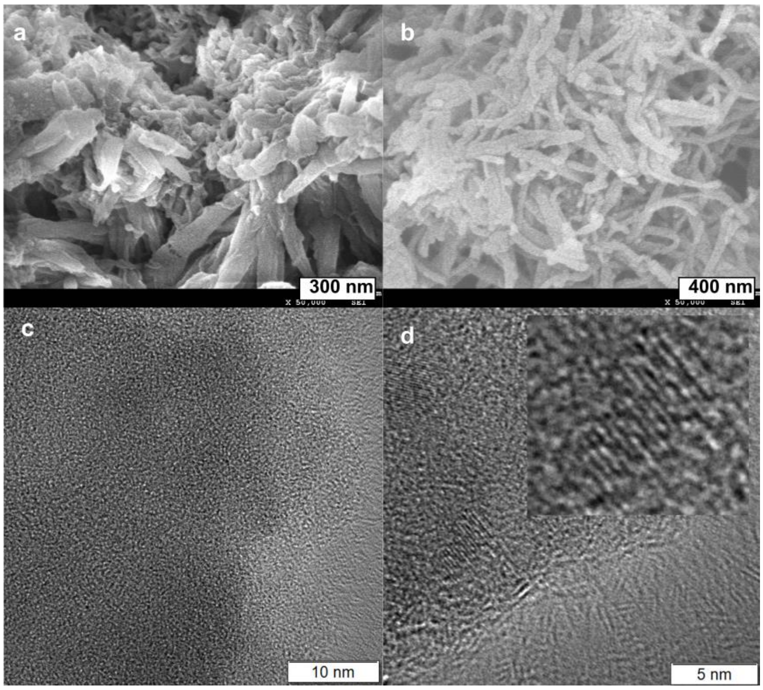

Figure S3 SEM and TEM images of AP-TfpBpy (a,c) and (b,d).COF-TfpBpy.

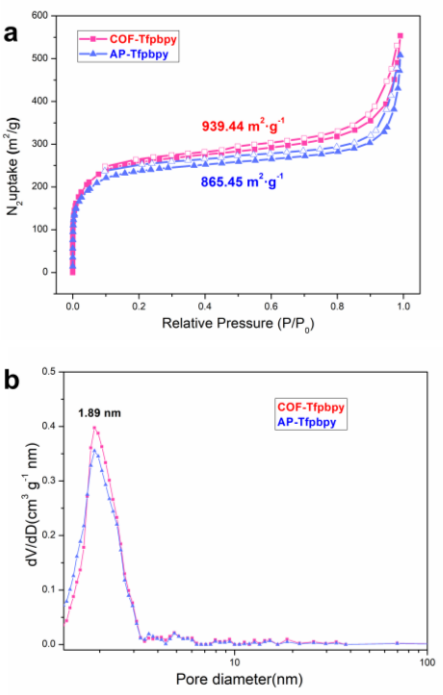

Figure S4 Adsorption and desorption curve (a) and porosity data (b) of COF-TfpBpy and AP-TfpBpy.

Note S1:

The powder X-ray diffraction (PXRD) pattern of COF-TfpBby (red line) shows that a strong peak corresponding to the (100) plane reflection is displayed at 2θ=3.68°,

indicating that there is an open channel. The broad peak at 2θ=26° is mainly due to the π-π accumulation between the COF layers corresponding to the (002) plane. The relatively broad peaks can be attributed to strain defects and grain size effects in the crystal lattice. However, AP-TfpBpy (green line) has no obvious diffraction peak at 2θ=3.68°, indicating that its structure has no crystal form (**Figure S1a**). Based on the experimental and simulated XRD pattern, COF-TfpBpy should be a COF materials with AA stacking mode rather than conjugated porous polymer.

According to FT-IR (**Figure S1b**), strong peaks corresponding to the stretching frequency of the ketone form are observed at 1608 cm^{-1} (C=O) and 1579 cm^{-1} (C=C) **(Figure S1b**). It is the same as the COF-TfpBpy reported in the previous literature. AP-TfpBpy also has two corresponding strong peaks at 1607 cm^{-1} and 1579 cm^{-1}, indicating that C=O and C=C also exist, and the other parts may be different in infrared due to crystallinity.

As shown in **Figure S1c**, the ^{13}C CP-MAS solid state NMR of COF-TfpBpy shows carbonyl (C=O), imine bond (C=N) and carbon at δ182.8, 148.9 ppm and 106.0 ppm respectively. Carbon double bond (C=C) carbon signal. The ^{13}C CP-MAS solid-state NMR of AP-TfpBpy also has a corresponding peak at the same position. The solution ^{13}C NMR (**Figure S1c, d**) spectra of the two structures are not significantly different, indicating that they belong to the same substance in structure.

The N1 s deconvolution of COF-TfpBpy and AP-TfpBpy showed two peaks at 398.35 eV and 399.59 eV, corresponding to the N atoms of pyridine N and secondary N (**Figure S2c**); their C1 s deconvolution are 285.6 and 284.3 eV, corresponding to the hydroxyl and carbonyl groups on the benzene ring, respectively (**Figure S2b**). Their O1 deconvolutions are 532.1 and 530.5 eV, respectively, corresponding to C-OH and C=O on the benzene ring, which may be caused by the existence of tautomerism(**Figure S2d**).

The SEM and TEM images (**Figure S3**) show that the crystals of COF-TfpBpy and AP-TfpBpy are both interlaced linear morphology, and there are a large number of individual crystallites aggregated; and they seem to be only a few layers thick. However, it can be seen from the SEM image that COF-TfpBpy has a tendency to form a spherical structure, but AP-TpBpy does not.

The adsorption and desorption curves and BET of COF-TfpBby and AP-TfpBpy under N_2 are shown in **Figure S4**. It is known that the specific surface areas of COF-TfpBby and AP-TfpBpy are 939.44 m^2 g^{-1} and 865.45 m^2 g^{-1}, respectively.

AP-TfpBpy's Brunauer-Emmett-Teller (BET) is significantly reduced, indicating that the crystallinity of COFs directly affects the size of its active contact surface.[6] The pore size distribution graph of COF-TfpBpy and AP-TfpBpy prepared by solvothermal method has a peak at 1.89 nm, indicating that the material is a mesoporous material.

Part 3 Characterizations of g-C_3N_4

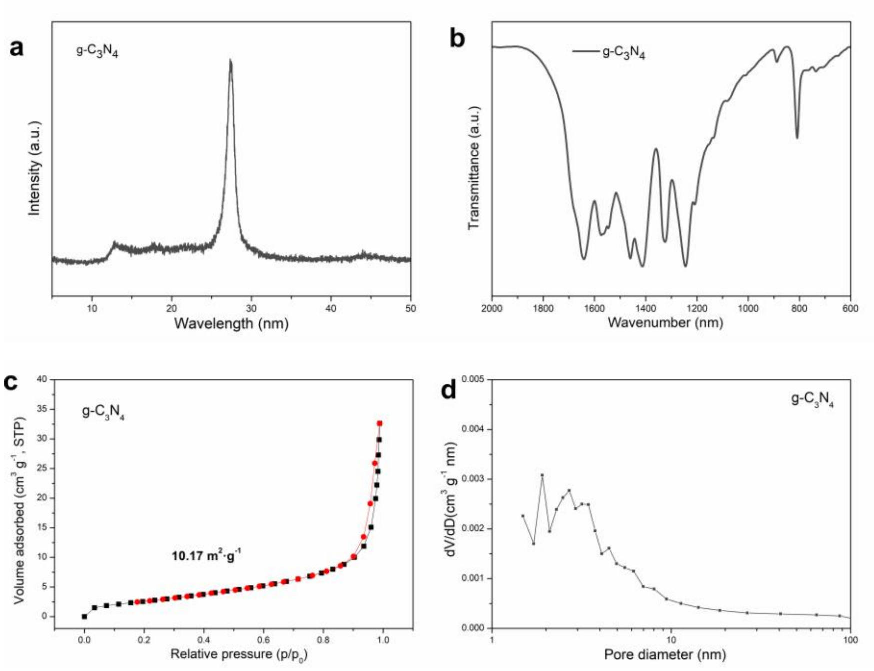

Figure S5 Structural characterization of g-C_3N_4. (a) XRD pattern of g-C_3N_4; (b) IR spectra of g-C_3N_4; (c and d) adsorption and desorption curve and porosity data of g-C_3N_4.

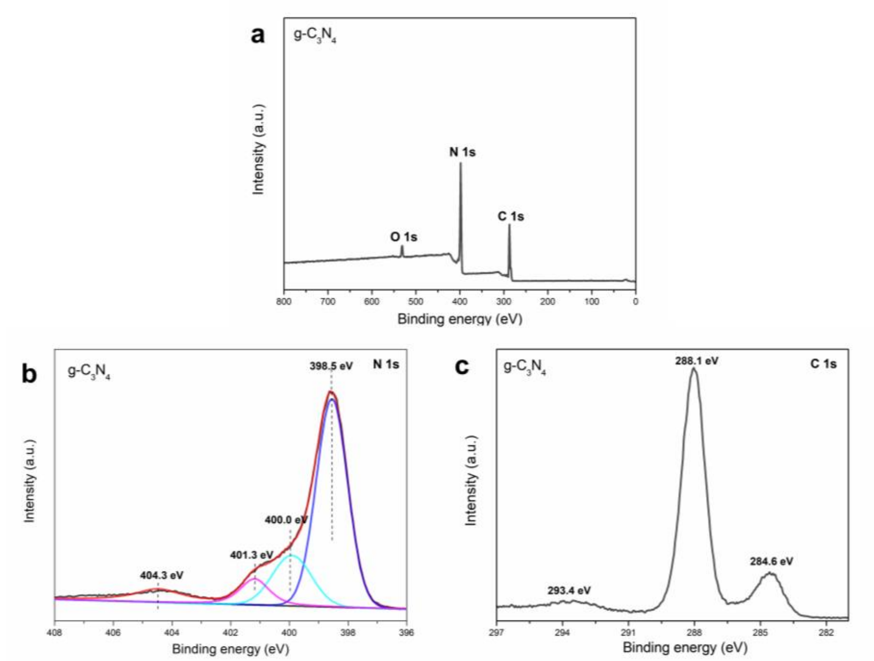

Figure S6 The XPS spectrum of g-C_3N_4. Survey scan XPS profile (a), N 1s (b), and C 1s (c) XPS spectrum of g-C_3N_4.

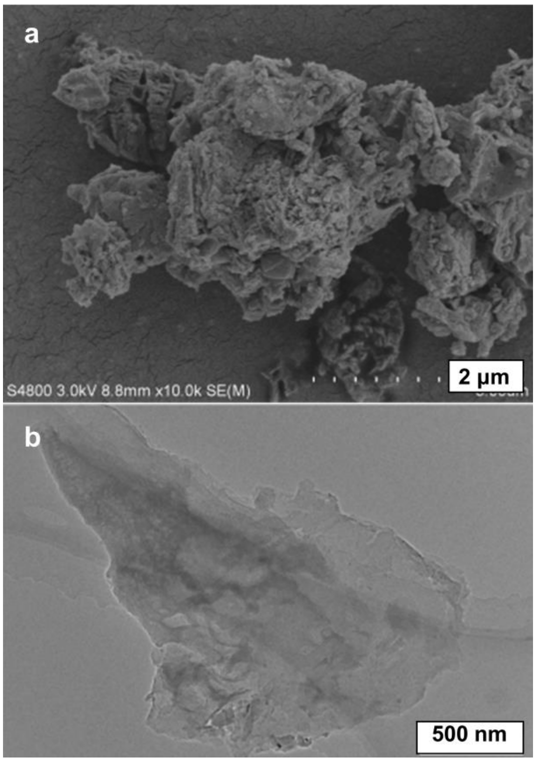

Figure S7 SEM (a) and TEM (b) images of g-C_3N_4.

Note S2:

Figure S5a shows that the XRD spectrum of g-C_3N_4 has two peaks at 12.7° and 28°, which are consistent with the typical graphite phase carbon nitride diffraction peaks, corresponding to the (100) peak of the in-plane repeating unit and stacked between the aromatic ring layer (002) peak, which means that g-C_3N_4 is obtained.

The FTIR spectrum of g-C_3N_4 is shown in **Figure S5b**. The characteristic peak at 2900-3400 cm^{-1} corresponds to the stretching vibration absorption peak of -NH_2 or -OH, the peak in the range of 1200-1650 cm^{-1} corresponds to the stretching vibration of the CN heterocyclic ring, and the peak at 810 cm^{-1} is the characteristic peak of the triazine ring structure.[7]

The g-C_3N_4 prepared by thermal polymerization has a small specific surface area, low catalytic efficiency, and lack of active sites. As shown in **Figure S5**. the Brunauer-Emmett-Teller (BET) specific surface area of traditional synthetic g-C_3N_4 is 10.17 m^2 g^{-1}.

XPS spectroscopy confirms that there are only three elements of C, O and N in the material, and there are three different bonding environments for C and four different bonding environments for N, which further confirms the successful preparation of g-C_3N_4 (**Figure S6a**).

The N 1s spectrum of g-C_3N_4 (**Figure S6b**) can be divided into 4 characteristic peaks. The binding energies are 398.5 eV, 400.0 eV, 401.3 eV and 404.3 eV, which correspond to the sp^2 hybrid N in triazine (N-C=N), Tertiary N in (N-$(C)_3$), N in C-NH_2 structure, and charge effects, respectively.

The C 1s spectrum of g-C_3N_4 (**Figure S6c**) can be divided into 3 characteristic peaks. The characteristic peaks with binding energies of 284.6 eV and 288.1 eV are attributed to sp^2 C=C bond and sp^2 bonded carbon (N-C=N), respectively; The weak peak at 293.4 eV may be due to the O-C=O bond or C=O bond generated by g-C_3N_4 heating in air. [8]

As shown in **Figure S7**, it can be seen from the scanning electron microscope (SEM) that g-C_3N_4 is a large layered structure, and the transmission electron microscope (TEM) image shows that its thickness is relatively thin.

Part 4 Standard curve of H_2O_2

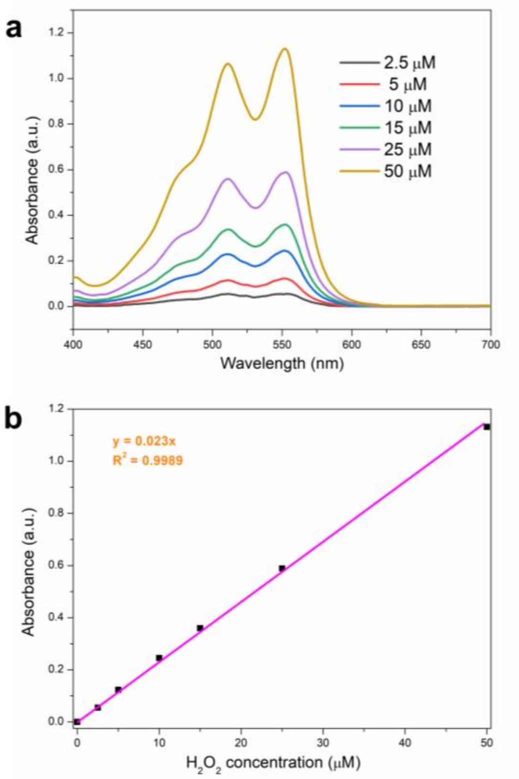

Figure S8 (a) The absorption spectra of H_2O_2 at different concentrations (0 µM, 2.5 µM, 5 µM, 10 µM, 15 µM, 25 µM, 50 µM) at wavelengths of 400-700 nm; (b) and a standard curve of H_2O_2 concentration-absorbance was drawn.

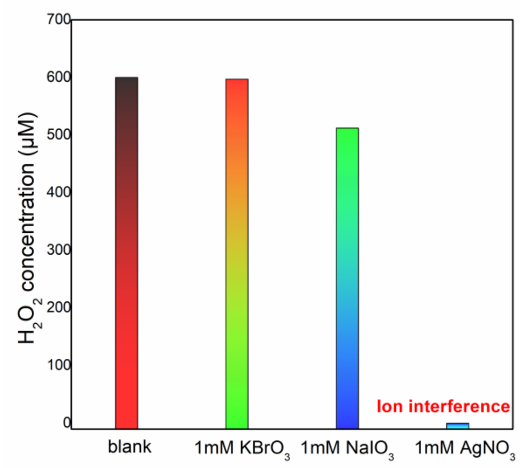

Figure S9 The effect of different electron sacrificial reagents on DPD-POD method to measure H_2O_2 concentration.

Note S3:

30% H_2O_2 standard solution is used to prepare H_2O_2 solutions of different concentrations. The absorbance is measured under an ultraviolet-visible

spectrophotometer (**Figure S8**).

Bader proposed the DPD (N,N-diethylp phenylened ianine)/POD (horserad ish pemxidase) method to measure low-concentration hydrogen peroxide in water in 1988. The experimental method is: adjust the pH of the sample, and then add DPD and POD solutions. If there is hydrogen peroxide in the water, the hydrogen peroxide will oxidize POD, and the oxidation product of POD will then oxidize DPD into positive ion radical DPD $\bullet^{+}$. DPD $\bullet^{+}$ is a pink compound, it has two absorption peaks, 510 mm and 551 mm. Because this method can only detect low-concentration hydrogen peroxide in water, and our photocatalytic production of hydrogen peroxide has a higher concentration, it must be diluted before testing.[9]

In order to detect the influence of different electronic sacrificial reagents on the detection method, as shown in **Figure S9**, we set it under dark conditions, use the same concentration of hydrogen peroxide, and add the same concentration of electronic sacrificial reagents ($KBrO_3$, $NaIO_3$, $AgNO_3$) to Judge their influence on the concentration of H_2O_2 measured by the DPD-POD method. It can be clearly seen from the figure that $KBrO_3$ has the least impact on the detection method, while $AgNO_3$ has the opposite effect. In summary, we chose $KBrO_3$ as the electronic sacrificial reagent.

Part 5 Catalytic activity and stability

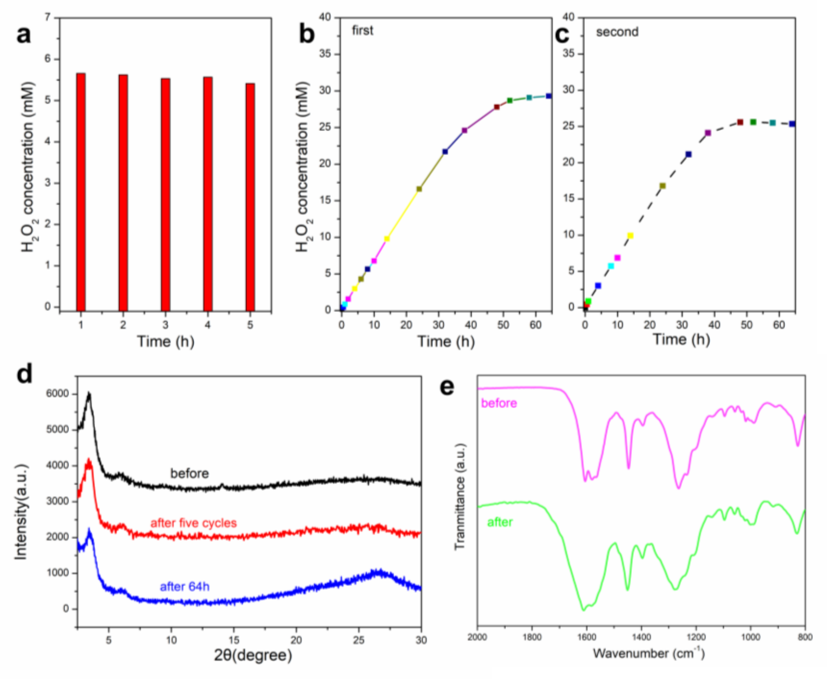

Figure S10 (a) Five cycles of COF-TfpBpy after 8 h irradiation for photocatalytic H_2O_2 production. (b and c) long time photocatalytic synthesis of H_2O_2. (d) XRD of COF-TfpBpy before and after photocatalytic reaction. (e) IR spectra of COF-TfpBpy before and after photocatalytic reaction . Conditions: $\lambda > 420$ nm; at 298 K under one sun light illumination (100 mW cm^{-2}); 400 mL H_2O; 600 mg COF-TfpBpy.

Part 6 AYQ and SCC

The photocatalytic reaction was carried out in pure deionized water (400 ml) with photocatalyst (600 mg) in a foil reflective light-concentrating reactor. After ultrasonication and Air bubbling, the bottle was irradiated by an Xe lamp (light intensity at 420-700 nm: 40.8 mW cm^{-2}). The optical power was determined by a PL-MW 2000 photoradiometer (Beijing Perfect Light Technology Co., Ltd., Beijing, China).

For Calculation of the apparent quantum yield (AQY), the incident light was monochromated by band-pass glass filters.

For solar-to-chemical conversion (SCC), a UV cut filter (λ> 420 nm) was used to avoid decomposition of the formed H_2O_2 by absorbing UV light.

Calculation of the apparent quantum yield (AQY):

$$\mathrm{AQY}=\frac{N_{H_2O_2}}{N_P}$$

$$=\frac{2\times \text{the number of evolved } \mathrm{H_2O_2} \text{ molecules}}{\text{the number of incident photons}}\times 100\%$$

$$=\frac{2N_a\times M_{H_2O_2}}{\frac{PSt\lambda}{hc}}$$

$$=\frac{2\times 6.02\times 10^{23}\times M_{H_2O_2}}{\frac{PS\times 40\times 60\times 420\times 10^{-9}}{6.626\times 10^{-34}\times 3\times 10^{8}}}$$

$$\text{SCC efficiency}(\%)=\frac{\left[\Delta\text{G for } H_2O_2 \text{ generation}\left(\text{J } mol^{-1}\right)\right]\left[H_2O_2 \text{ formed}(\text{mol})\right]}{\left[\text{total input power }(\text{W})\right]\left[\text{reaction time}(\text{s})\right]}\times 100\%$$

where ΔG= 117 kJ mol^{-1}. For example, when using COF-TpBpy as the catalyst, the irradiated sample areas are 16 cm^2 during 60 min of on sun illumination. Therefore, the calculated total input energy is 5760 J. During the 60 min photocatalytic reaction, x μmol H_2O_2 is generated and the energy generated by H_2O_2 formed is 0.117x J.

SCC Efficiency=0.117x/5760

Table S1. Comparison of H_2O_2 production of polymer photocatalytic material under one sun light when non-sacrificial/stabilizer-free. Light intensity: AM 1.5 100 mW cm^{-2}

Photocatalysts	Light source	H_2O_2 (μM h^{-1})	AQY (%)@420 nm	SCC (%)	Temperature (K)	Refs.
**$g\text{-}C_3N_4$/PDI**	λ>420 nm	~190	2.5	~0.1	298	10
**$g\text{-}C_3N_4$/BDI**	λ>420 nm	~250	4.6%	0.13	298	11
**$g\text{-}C_3N_4$/PDI/rGO**	λ>420 nm	~380	6.1	0.2	298	12
**$g\text{-}C_3N_4$/MTI**	λ >420 nm	~330	6.1	0.18	298	13
**$g\text{-}C_3N_4$ /PDI-BN-rGO**	λ >420 nm	530	7.3	0.28	298	14
**CTF**	λ>420 nm	58.4		0.14	298	15
**RF resins[a]**	λ>420 nm	~1080	7.5[d]	0.5	333	16
**RF resins[b]**	λ>420 nm	~1350	8.4	~0.75	333	17
**RF resins[c]**	λ>300 nm	~1480		1.07	323	18
**RF/P3HT**	λ>300 nm	~1840	10	1.0	333	19
**COF-TfpBpy**	λ >420 nm	1042	8.1	0.57	298	This work
	λ >300 nm	1970	13.5	1.08	333	

[a] Prepared by Stöber method
[b] Prepared by acid-catalysed polycondensation
[b] Mesoporous resorcinol–formaldehyde spheres (MRFS)
[d] @450 nm

Part 7 Band structure of g-C_3N_4, COF-TfpBpy and AP-TfpBpy

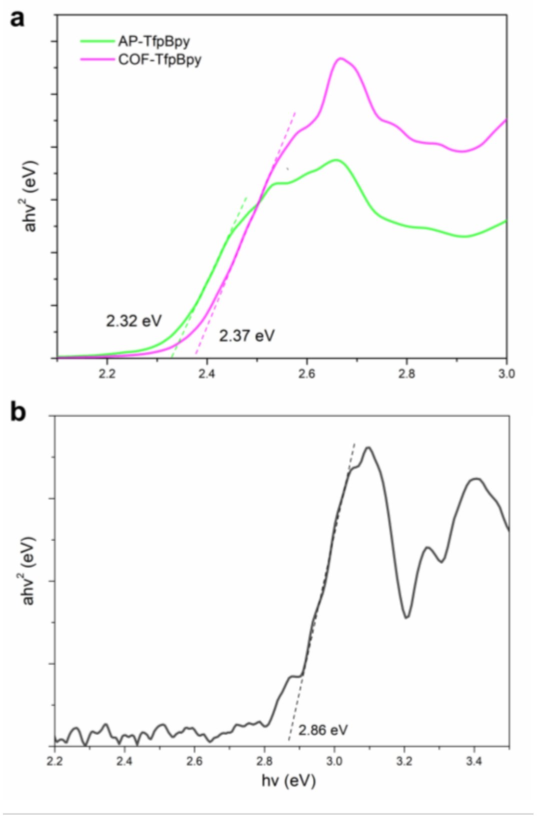

Figure S11 Kubelka-Munk-transformed reflectance spectra of COF-TfpBpy, AP-TfpBpy (a) and g-C_3N_4 (b).

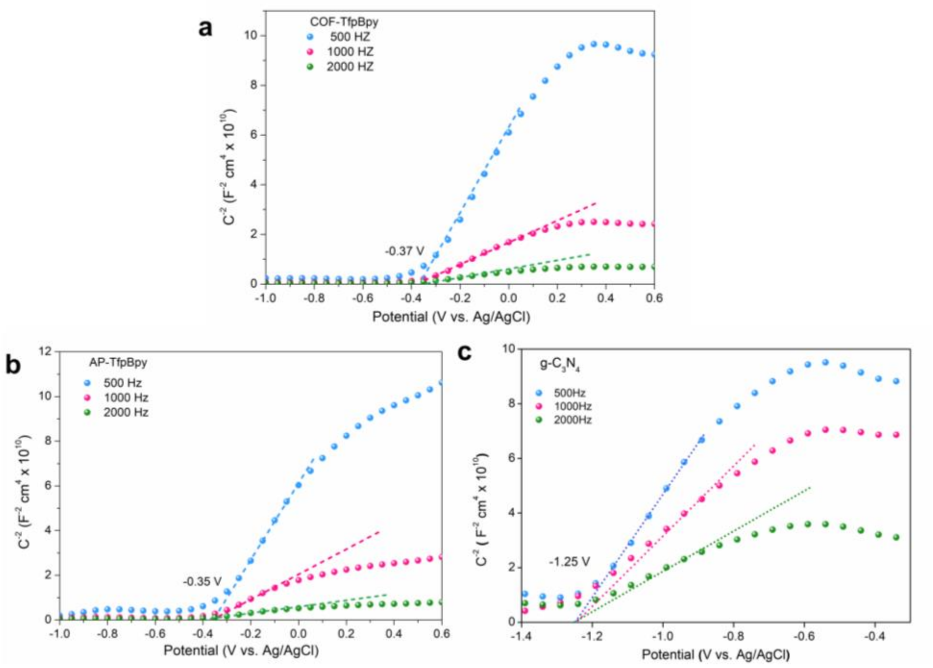

Figure S12 Mott-Schottky plots of COF-TfpBpy (a), AP-TfpBpy (b) and g-C_3N_4 (c) of film electrodes at frequency of 500 Hz, 1000 Hz, and 2000 Hz in an aqueous solution of Na_2SO_4 (0.1 M; pH 6.6).

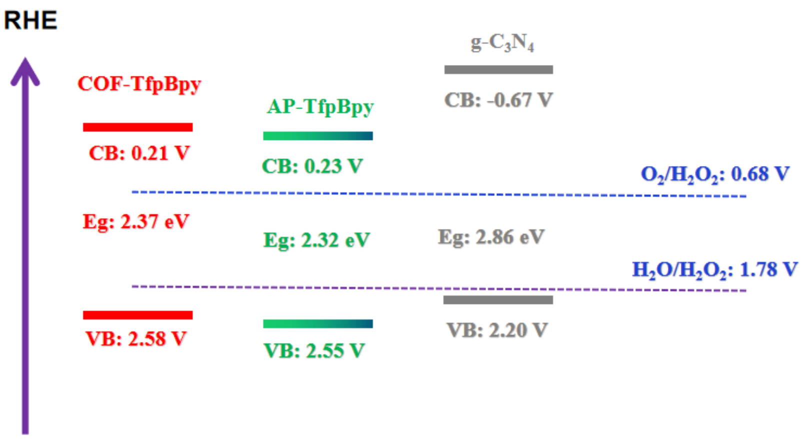

Figure S13 Schematic band structure diagram of COF-TfpBpy, AP-TfpBpy and g-C_3N_4.

Note S4:

Kubelka-Munk equation : $F(R_\infty) = \frac{K}{S} = \frac{(1-R_\infty)^2}{2R_\infty}$

K- Absorption coefficient，S- Scattering coefficient

R- The limit value of the reflection coefficient R_∞ of an infinitely thick sample $F(R_\infty)$- Reduction function or Kubelka-Munk function

The actual measured R_∞' is not the absolute reflectance R_∞, that is, the relative reflectance relative to a standard sample.

The optical bandgaps estimated from the corresponding Kubelka–Munk-transformed reflectance spectra are 2.37 eV, 2.32 eV, and 2.86 eV for COF-TfpBpy, AP-TfpBpy and g-C_3N_4, respectively (**Figure S11)**. Mott-Schottky plots showed the CB values of COF-TfpBpy, AP-TfpBpy and g-C_3N_4 are 0.21, 0.23 and -0.67 V (**Figure S12**). According to the energy band structure diagram, the VB values of COF-TfpBpy, AP-TfpBpy and g-C_3N_4 are 2.58, 2.55 and 2.20 V (**Figure S13)**. The band structure of COF-TfpBpy and AP-TfpBpy is sufficient for the synthesis of H_2O_2 from H_2O ($E_{H2O2/H2O}$ = +1.78 V vs NHE) and O_2 ($E_{O2/H2O2}$ = +0.68 V vs NHE)

Part 8 Wettability of the COF-TfpBpy

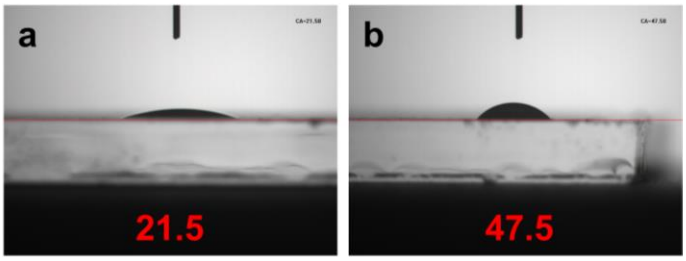

Figure S14 Contact angle of COF-TfpBpy and g-C_3N_4.

Part 9 Characterizations of COF-TfpDaaq

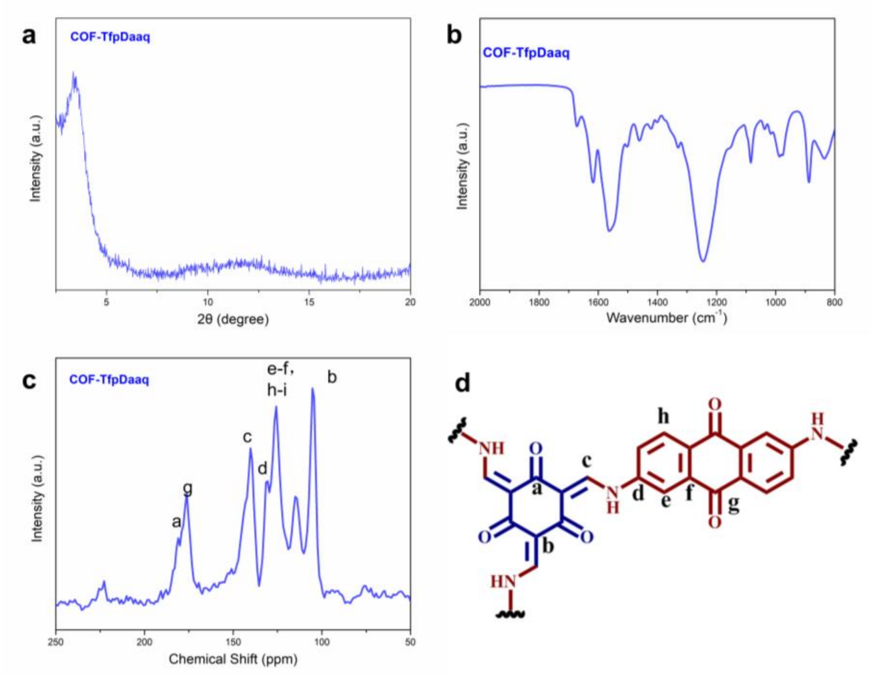

Figure S15 The XRD (a), IR (b) and Solution ^{13}C NMR (c, d) spectra of COF-TfpDaaq.

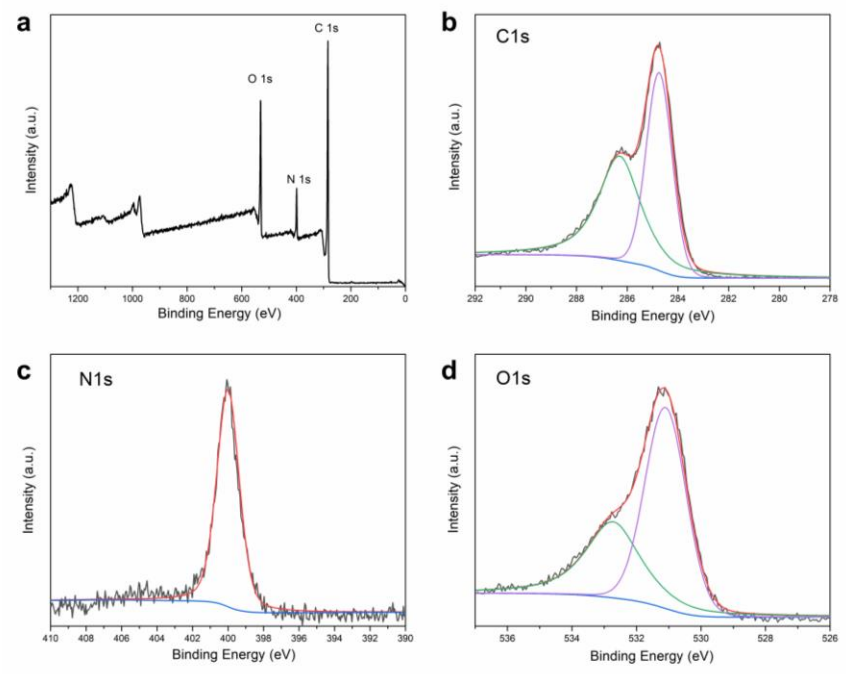

Figure S16 The XPS spectrum of COF-TfpDaaq. Survey scan XPS profile (a), C 1s (b), N 1s (c) and O 1s (d) XPS spectrum of COF-TfpDaaq.

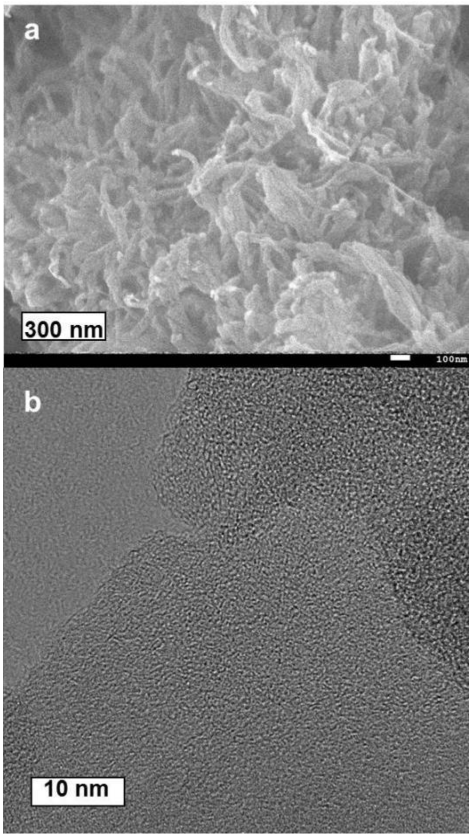

Figure S17 SEM (a) and TEM (b) images of COF-TfpDaaq.

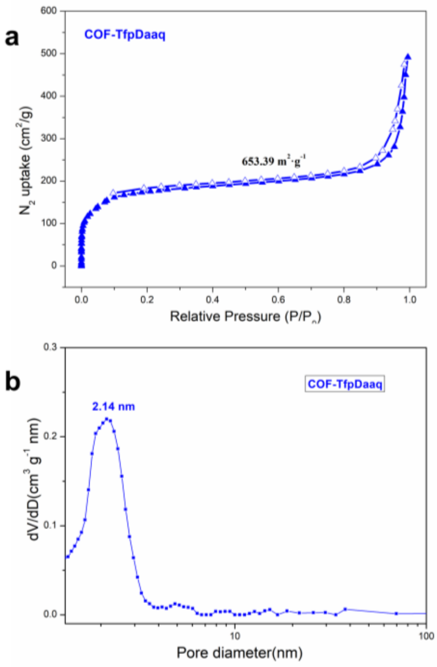

Figure S18 Adsorption and desorption curve (a) of porosity data (b) of COF-TfpDaaq.

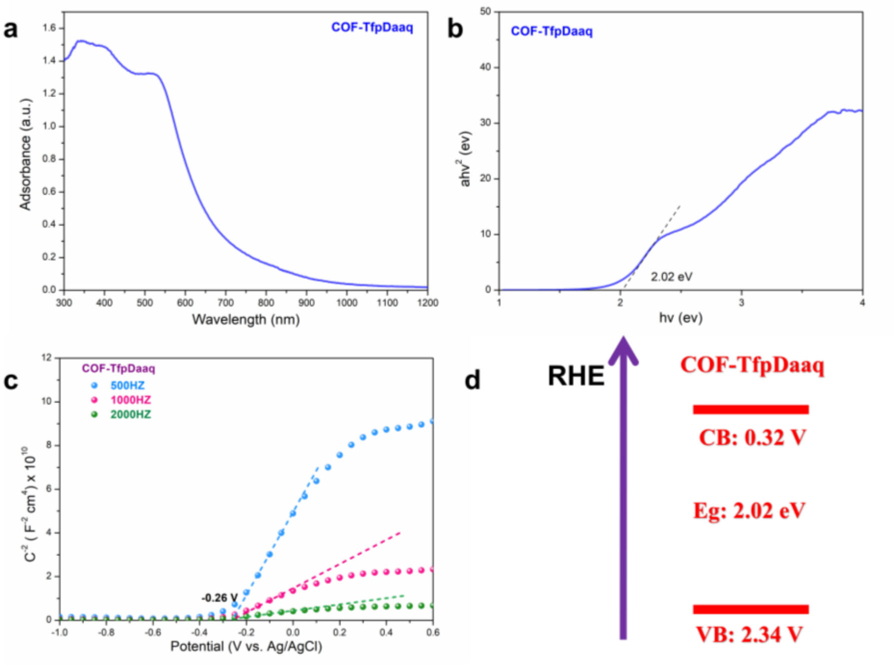

Figure S19 UV-vis DRS spectra (a), Kubelka-Munk-transformed reflectance spectra (b), Mott-Schottky plots (c) and schematic band structure diagram (d) of COF-TfpDaaq.

Note S5:

COF-TfpDaaq shows a strong diffraction peak of 3.5° and a broad peak of 27°, corresponding to the (100), and (001) reflection surfaces of the structure, respectively (**Figure S15a**). Fourier transform infrared (**Figure S15b**) and ^{13}C crosspolarization magic angle spinning (CP-MAS) solid-state NMR spectroscopy (**Figure S15c, d**) further confirms COF-TfpDaaq structures. The appearance of a new C-N stretching vibration peak at 1250 cm^{-1} is characteristic of β-ketoenamine C-N bond in COF-TfpDaaq.[20] The preferential formation of the keto form rather than the enol tautomer is further confirmed by the C=O stretching vibration at 1615 cm^{-1} and the absence of O-H bond resonance in its FT-IR spectrum. As the spectra of COF-TfpDaaq exhibit resonances at 145 ppm that are assigned to the enamine carbon (=CNH) and α-enamine carbon at 115 ppm. In addition, the resonance at ~180 ppm corresponds to the ketone resonance. XPS spectra show the presence of C, N, and O in COF-TfpDaaq (**Figure S16**). High-resolution X-ray photoelectron spectroscopy also differentiates between C, N and O atoms in the different bonding environments.[21] The morphology of as-prepared catalysts was characterized by Scanning Electron Microscope (SEM) and Transmission electron microscopy (TEM)

(**Figure S17**). According to SEM, it can be seen that COF-TfpDaaq is an interconnected porous network structure, and it also shows a structure similar to folds or wrinkles.

According to the COF-TfpBby N_2 adsorption and desorption curve and BET (**Figure S18**), it is known that its specific surface area is 653.39 m^2 g^{-1}. and the pore size distribution of COF-DAAQ has a peak at 2.14 nm, indicating that the material is a mesoporous material.

In addition, the energy band structure of COF-TfpDaaq and the positions of CB and VB are calculated by DRS spectra, Kubelka-Munk converted reflectance spectra and Mott-Schottky diagrams. It is found that the values of VB, CB and optical band gap correspond to 2.34, 0.32, 2.02 V (**Figure S19**).

Part 10 Characterizations of COF-TfpBd

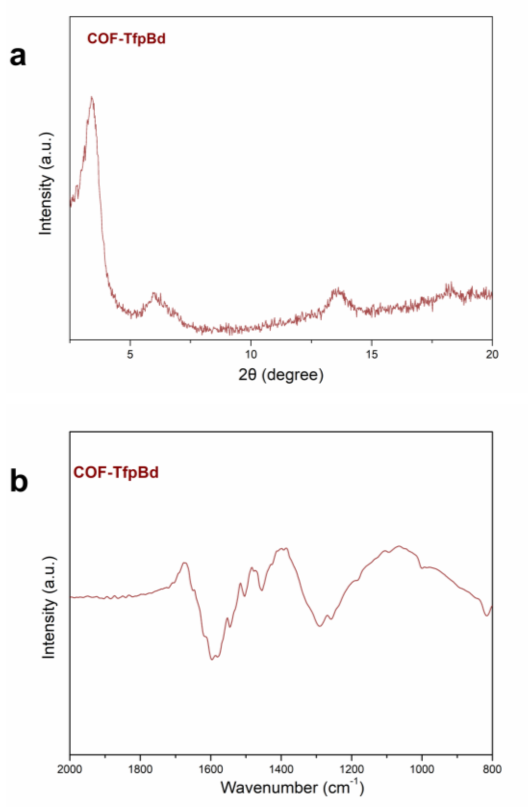

Figure S20 The XRD (a) and IR (b) spectra of COF-TfpBd.

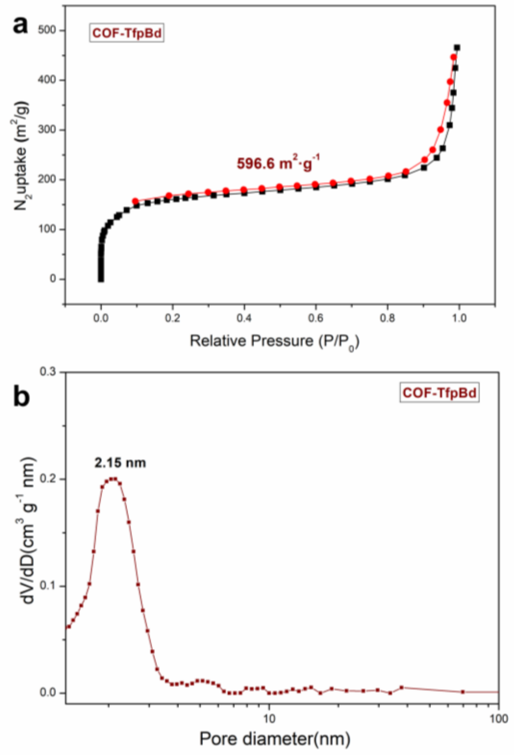

Figure S21 Adsorption and desorption curve (a) and porosity data (b) of COF-TfpBd.

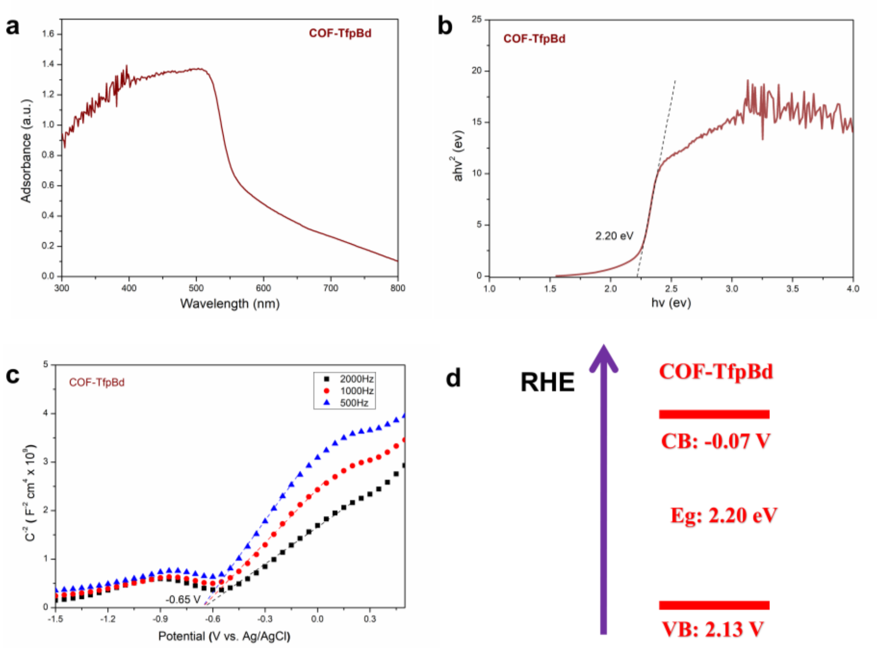

Figure S22 UV-vis DRS spectra (a), Kubelka-Munk-transformed reflectance spectra (b), Mott-Schottky plots (c) and schematic band structure diagram (d) of COF-TfpBd.

Note S6:

COF-TfpBD shows a strong diffraction peak of 3.4°, corresponding to the (100), reflection surfaces of the structure (**Figure S20a**). The FT-IR spectrum of COF-TfpBD (**Figure S20b**) shows a typical broad and strong band, which corresponds to the characteristic C=C stretch of aromatic hydrocarbons in the 1596 cm^{-1} region and the CN stretch of amines close to 1257 cm^{-1}. There is also a weak band close to 1618 cm^{-1}, which corresponds to the C=O bond of the ketone form in the tautomeric keto-enamine form.[22]

N_2 adsorption isotherm at 77 K to check the structural stiffness and permanent porosity of COF-TfpBD (**Figure S21**). The BET surface area of COF-TfpBD is 596.6 m^2 g^{-1}.The pore size distribution of COF-TfpBD was found to be between 2.15 nm.

In addition, the energy band structure of COF-TfpDaaq and the positions of CB and VB are calculated by DRS spectra, Kubelka-Munk converted reflectance spectra and Mott-Schottky diagrams. It is found that the values of VB, CB and optical band gap correspond to 2.13, -0.07, 2.20 V (**Figure S22**).

Part 11 Characterizations of COF-TfpPa

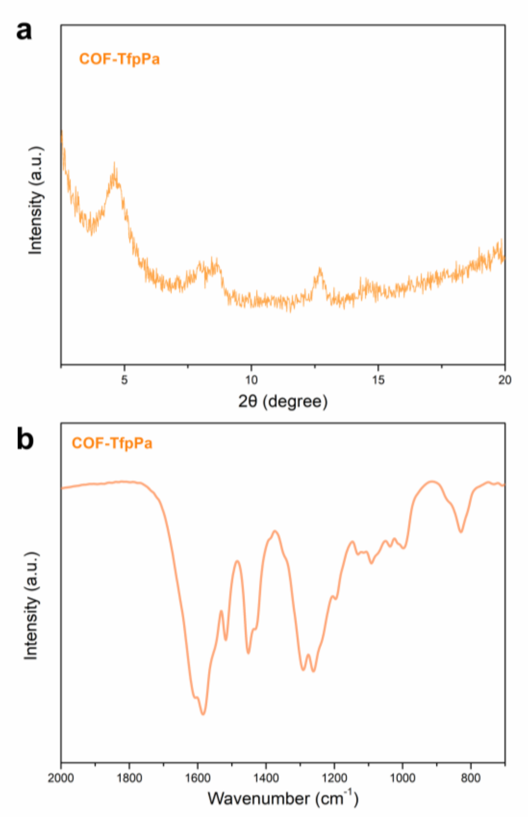

Figure S23 The XRD (a) and IR (b) spectra of COF-TfpPa.

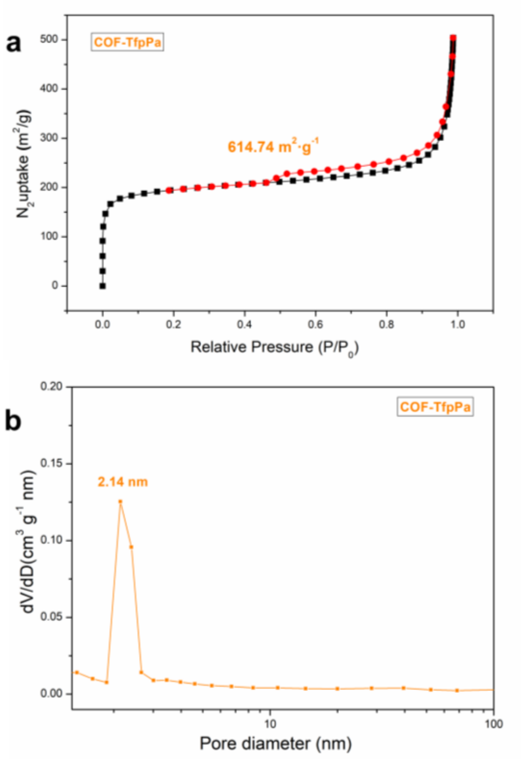

Figure S24 Adsorption and desorption curve (a) and porosity data (b) of COF-TfpPa.

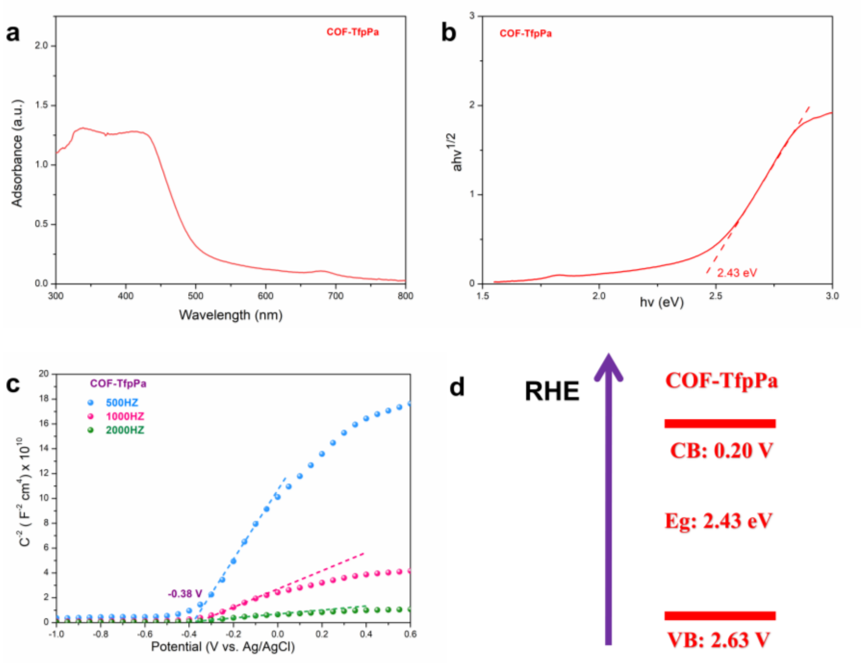

Figure S25 UV-vis DRS spectra (a), Kubelka-Munk-transformed reflectance spectra (b), Mott-Schottky plots (c) and schematic band structure diagram (d) of COF-TfpPa.

Note S7:

The powder X-ray diffraction (PXRD) pattern of COF-TfpPa shows a strong peak at 4.5°, corresponding to the reflection from the (100) plane (**Figure S23a**). There are also small peaks at 2θ=8.3°, 11.9° and 26.8° of COF-TfpPa, which are attributed to the (200), (210) and (001) reflection planes.[23]

Interestingly, the FT-IR spectrum does not show the characteristic stretch bands of hydroxyl (-OH) or imine (C=N) functional groups (**Figure S23b**). If the compound exists in the form of enol, it should be there. On the contrary, they present a strong peak under the peak at 1578 cm^{-1} in the ketone form, indicating that it exists in the ketone form. However, because of the peak broadening in the extended structure, the C=O peaks of COF-TfpPa at 1616 cm^{-1} are merged with the C-C stretching band at 1578 cm^{-1} and appeared as a shoulder. And the BET surface area of COF-TfpPa is 614.74 m^2 g^{-1}. The pore size distribution of COF-TfpBD was found to be between 2.14 nm (**Figure S24**).

In addition, the energy band structure of COF-TfpPa and the positions of CB and VB are calculated by DRS spectra, Kubelka-Munk converted reflectance spectra and Mott-Schottky diagrams. It is found that the values of VB, CB and optical band gap correspond to 2.63, 0.20, 2.43 V **(Figure S25)**.

Part 12 Characterizations of COF-TfpBpy-Mo

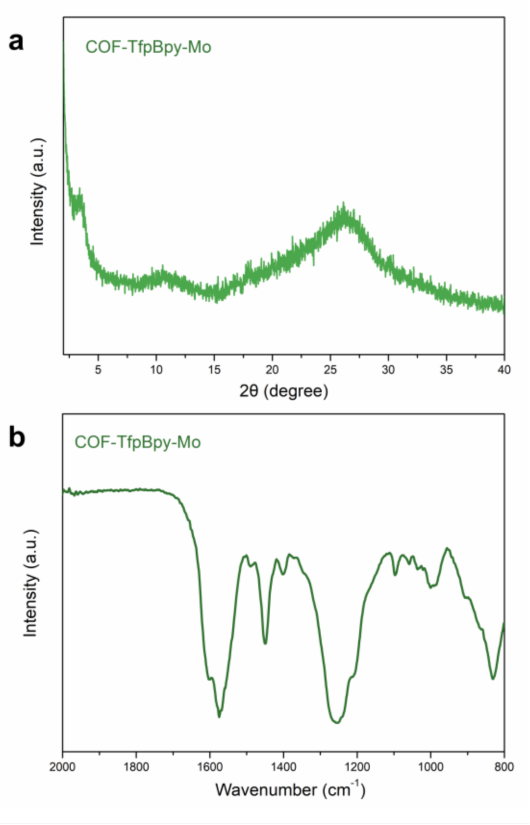

Figure S26 The XRD (a), and IR (b) spectra of COF- TfpBpy-Mo.

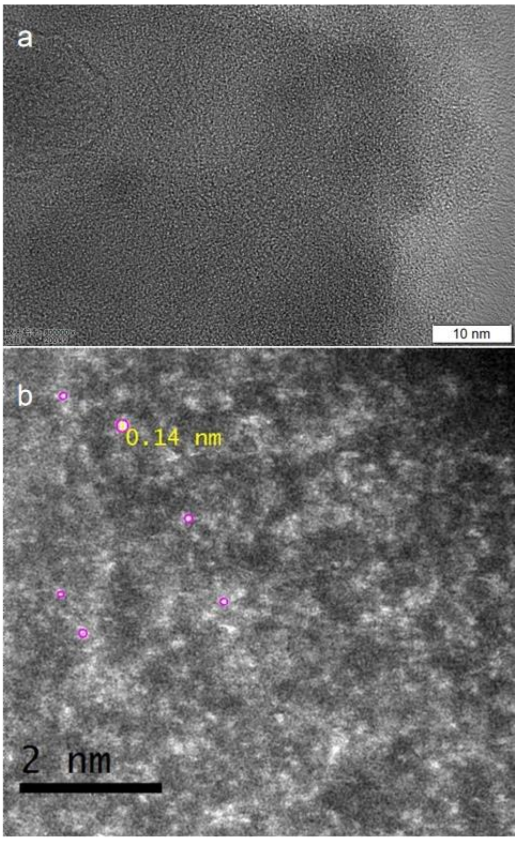

Figure S27 TEM (a) and HAAD-STEM (b) images of COF- TfpBpy-Mo.

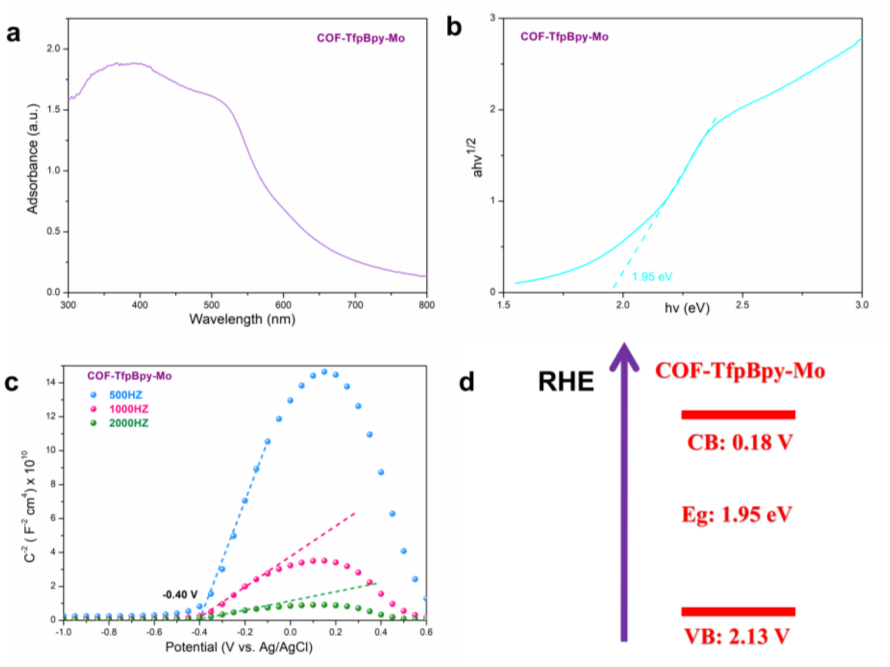

Figure S28 UV-vis DRS spectra (a), Kubelka-Munk-transformed reflectance spectra (b), Mott-Schottky plots (c) and schematic band structure diagram (d) of COF-TfpBpy-Mo.

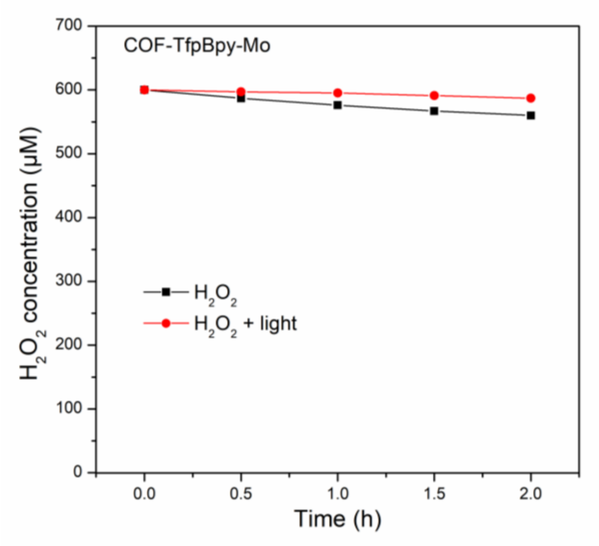

Figure S29 H_2O_2 degradation study by COF-TfpBpy-Mo. Conditions: $\lambda > 420$ nm (298K; xenon lamp, light intensity at 420-700 nm: 40.8 mW cm^{-2}), water (10 ml), catalyst (15 mg), Ar atmosphere.

Note S8:

According to the XRD patterns of COF-TfpBpy and COF-TfpBpy-Mo (**Figure S1a and Figure S26a**), it can be seen that there are no extra peaks, indicating that the

solid COF frame structure is still retained after the cobalt modification.

The FTIR spectrum shows that strong peaks corresponding to the stretching frequency of the ketone form are observed at 1608 cm^{-1} (C=O) and 1579 cm^{-1} (C=C) (**Figure S26b**). COF-TfpBpy-Mo still has the same characteristic functional groups as COF-TfpBpy, but some red shifts and obvious broadening of the C-N peak have occurred, which means that Mo and the bipyridine N atom are coordinated in the COF skeleton.

According to the transmission electron microscope (TEM) image (**Figure S27a**), COF-TfpBpy-Mo appears to be only a few layers thick. In the HAAD-STEM image (**Figure S27b**), it can be observed that Mo metal particles are present on the original COF-TfpBpy. TEM images showed that the morphology of COF was intact after loading Mo.

In addition, the energy band structure of COF-TfpBpy-Mo and the positions of CB and VB are calculated by DRS spectra, Kubelka-Munk converted reflectance spectra and Mott-Schottky diagrams. It is found that the values of VB, CB and optical band gap correspond to 2.13, 0.18, 1.95 V (**Figure S28**).

Part 13 Characterizations of AP-TfbBpy

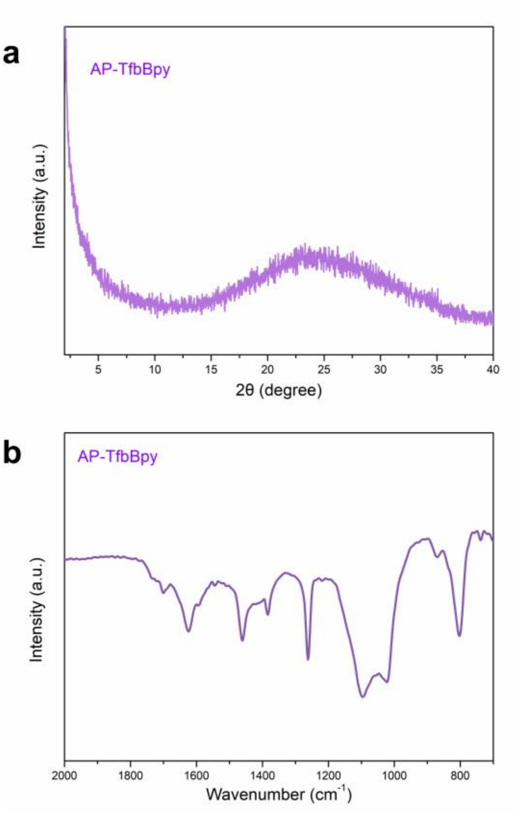

Figure S30 The XRD (a), and IR (b) spectra of AP-TfbBpy.

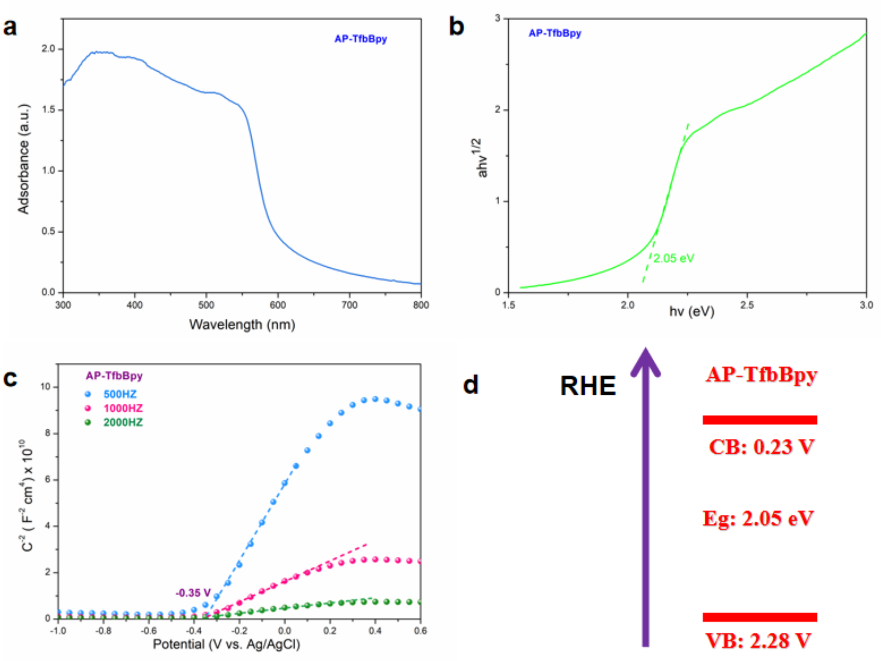

Figure S31 UV-vis DRS spectra (a) Kubelka-Munk-transformed reflectance spectra (b) Mott-Schottky plots (c) and schematic band structure diagram (d) of AP-TfbBpy.

Note S9:

The XRD pattern of AP-TfbBpy has a main diffraction peak at 2θ=24.5°, corresponding to the (001) plane; AP-TfbBpy has no obvious diffraction peak at 2θ=3.7°, indicating that its structure has no crystal form (**Figure S30a**).

The FT-IR spectrum of AP-TpbBpy shows a strong C=N stretch at 1618 cm^{-1} (**Figure S30b**), indicating the formation of an imine bond. The characteristic peak at 1271 cm^{-1} is attributed to the strong stretching of C-N. The new peak formed at 1584 cm-1 is designated as the C=C stretch, but the peak of AP-TfbBpy at this point is weakened to be negligible, indicating that TfbBpy does not exist in the ketone form.

In addition, the energy band structure of COF-TfbBpy and the positions of CB and VB are calculated by DRS spectra, Kubelka-Munk converted reflectance spectra and Mott-Schottky diagrams. It is found that the values of VB, CB and optical band gap correspond to 2.18, 0.13, 2.05 V (**Figure S31**).

Part 14 The influence of specific surface area and porosity

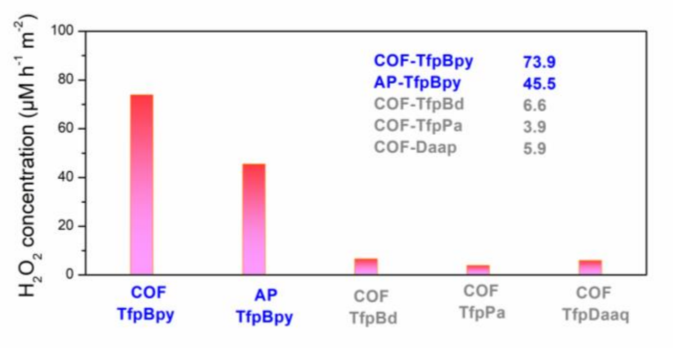

Figure S32 The amount of H_2O_2 generated per unit surface area of COF-TfpBpy, AP-TfpBpy, COF-TfpBd, COF-TfpPa and COF-TfpDaaq.

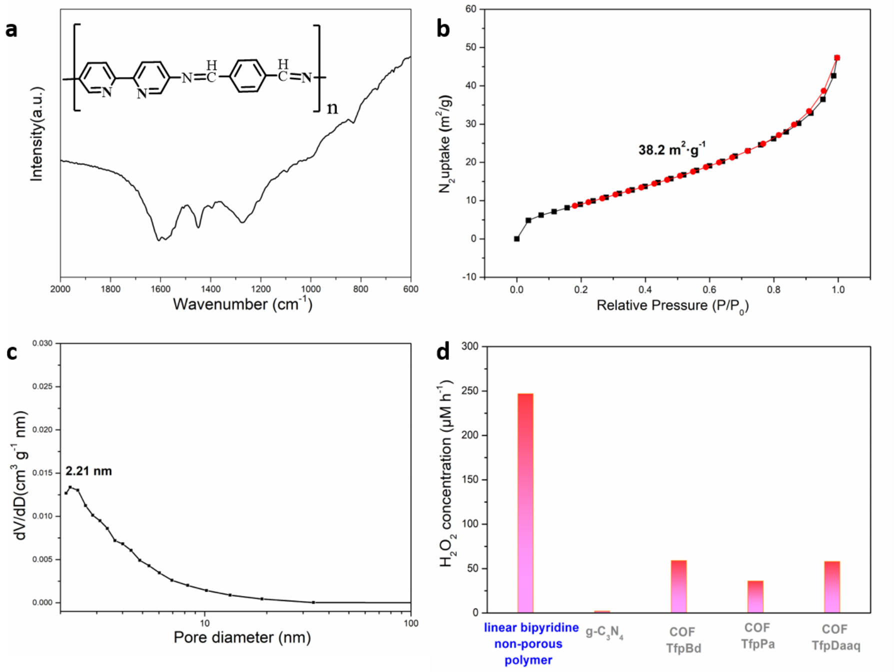

Figure S33 Linear bipyridine non-porous polymer: (a) structure and IR spectra, (b) BET, (c) porosity data, (d) photocatalytic activity for H_2O_2 production in pure water.

Part 15 Average electron transfer number of ORR

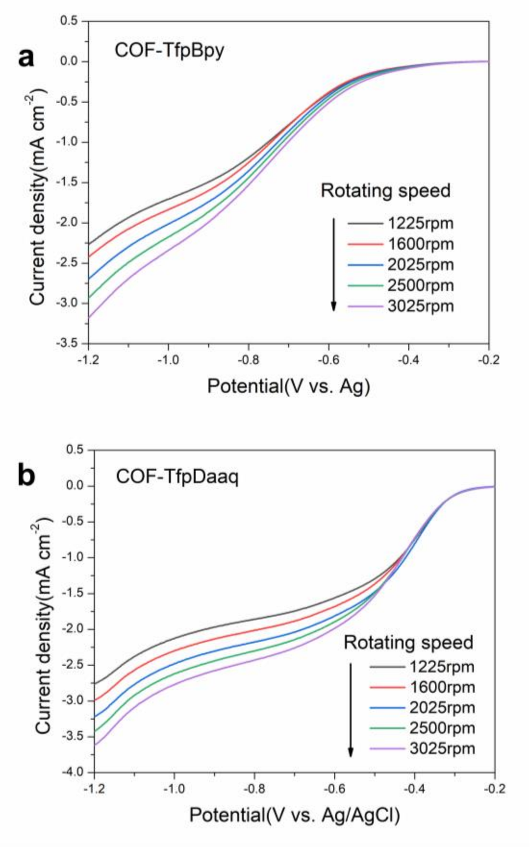

Figure S34 Linear-sweep RDE voltammograms of (a) COF-TfpBpy and (b) COF-TfpDaaq measured at different rotating speeds.

Part 16 ESR and Capture experiment of active species

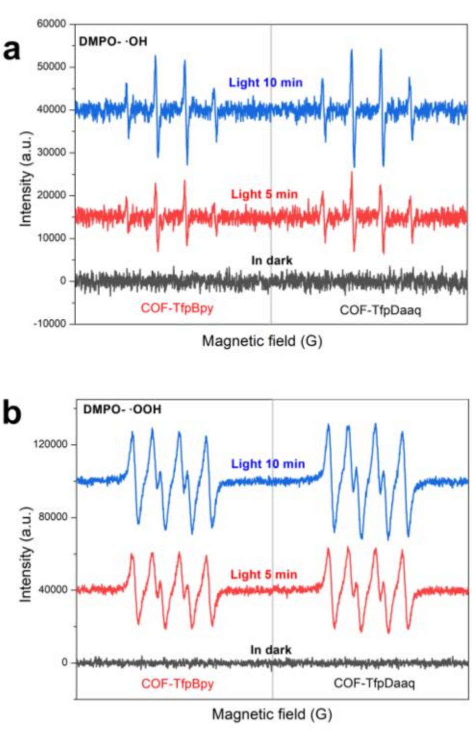

Figure S35 Under dark and visible light, the DMPO spin trapping EPR spectra of COF-TfpBpy and COF-TfpDaaq measured ·OH (a) and ·OOH (b).

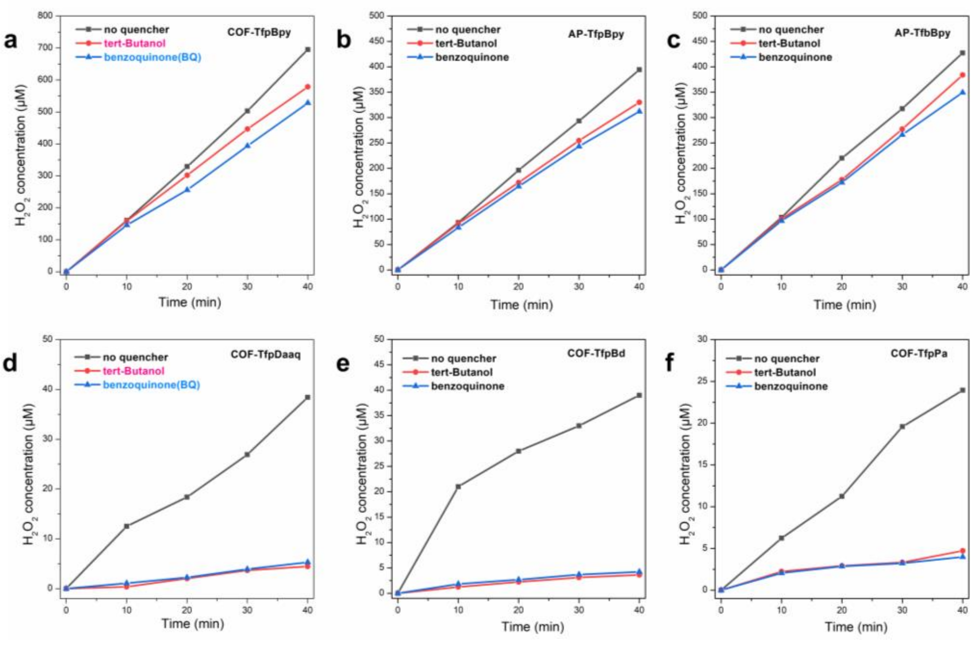

Figure S36 The influence of benzoquinone (BQ, $\cdot O_2^-$ scavenger) and tert Butanol (·OH scavenger) for the H_2O_2 generation on bipyridine based COF-TfpBpy, AP-TfbBpy and AP-TfpBpy, and non-bipyridine based COF-TfpDaaq, COF-TfpBd and COF-TfpPa.

Part 17 In-situ Fourier transform infrared (in situ IR)

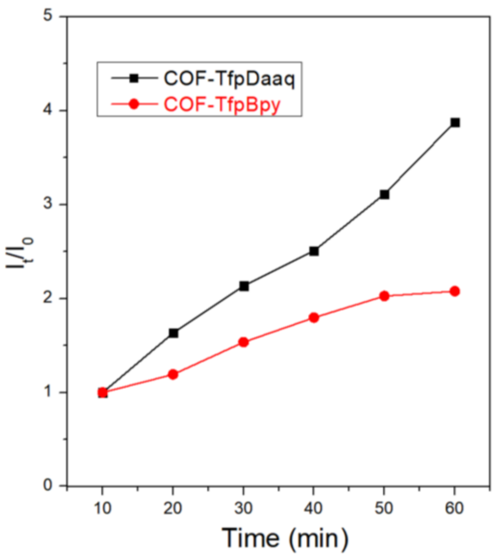

Figure S37 C=N functional group change graph over time.

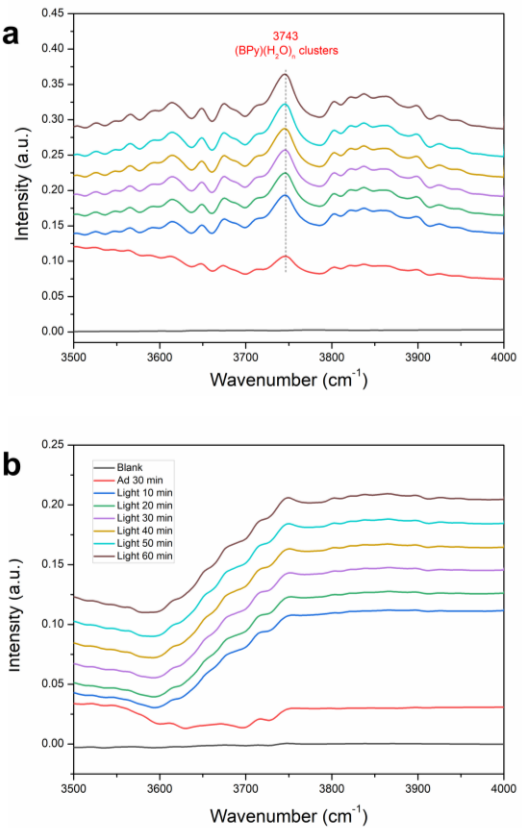

Figure S38 In-situ FT-IR spectrum of (a) COF-TfpBpy (b) COF-TfpDaaq for photosynthetic H_2O_2 production at 3500-4000 cm^{-1}.

Part 18 The importance of protonation for H_2O_2 Photosynthesis.

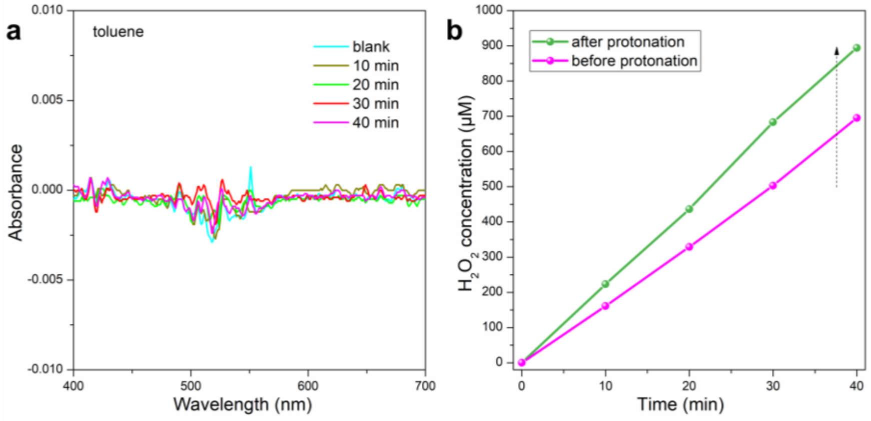

Figure S39 (a) The absorption spectra of H_2O_2 photosynthesis in nonaqueous solvents; (b) protonation of COF-TfpBpy for H_2O_2 photosynthesis. Conditions: $\lambda > 420$ nm (298K; xenon lamp, light intensity at 420-700 nm: 40.8 mW cm^{-2}), water (10 ml), catalyst (15 mg).

Part 19 Theoretical calculation

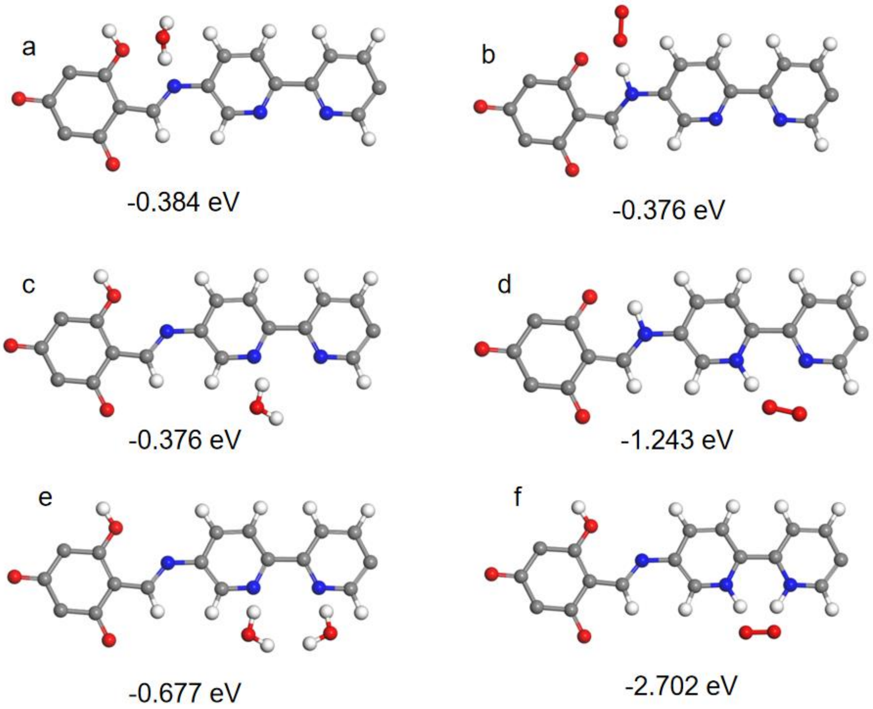

Figure S40 Adsorption energies of H_2O and O_2 on different N sites of COF-TfpBpy. (a) one H_2O molecular on imine nitrogen atom, (b) O_2 molecular on bipyridine on C=NH$^+$ via O_2^- intermediate species, (c) one H_2O molecular on on bipyridine nitrogen atoms, (d) O_2 molecular on bipyridine via O_2^- intermediate species, (e) two H_2O molecular on bipyridine nitrogen atoms, (f) O_2 molecular on bipyridine via endoperoxide intermediate specie.

Part 20 References

(1) H. B. Aiyappa, J. Thote, D. B. Shinde, R. Banerjee, S. Kurungot, Cobalt-Modified Covalent Organic Framework as a Robust Water Oxidation Electrocatalyst, *Chemistry of Materials. 2016, 28, 4375-4379.*

(2) J. Y. Zhang, G. Zhang, S. Y. Jin, Y. J. Zhou, Q. H. Ji, H. C. Lan, H. J. Liu, J. H. Qu, Graphitic N in nitrogen-Doped carbon promotes hydrogen peroxide synthesis from electrocatalytic oxygen reduction, *Carbon. 2020, 163, 154-161.*

(3) S. Kandambeth, A. Mallick, B. Lukose, M. V. Mane, T. Heine, R. Banerjee, Construction of Crystalline 2D Covalent Organic Frameworks with Remarkable Chemical (Acid/Base) Stability via a Combined Reversible and Irreversible Route, *Journal of the American Chemical Society. 2012, 134, 19524-19527.*

(4) K. Hu, Y. X. Lv, F. G. Ye, T. Chen, S. L. Zhao, Boric Acid Functionalized Covalent-Organic Framework for Specific Enrichment and Direct Detection of Cis-Diol-Containing Compounds by Matrix-Assisted Laser Desorption/ Ionization Time-of-Flight Mass Spectrometry, *Analytical chemistry. 2019, 91, 6353-6362.*

(5) N. Lu, N. Liu, Y. Hui, K. F. Shang, N. Jiang, J. Li, Y. Wu, Characterization of highly effective plasma-treated g-C_3N_4 and application to the photocatalytic H_2O_2 production, *Chemosphere. 2020, 241, 124927.*

(6) D. B. Shinde, H. B. Aiyappa, M. Bhadra, B. P. Biswal, P. Wadge, S. Kandambeth, B. Garai, T. Kundu, S. Kurungot, R. Banerjee, A mechanochemically synthesized covalent organic framework as a proton-conducting solid electrolyte, *Journal of Materials Chemistry A. 2016, 4, 2682-2690.*

(7) S. Bellamkonda, R. Shanmugam, R. R. Gangavarapu, Extending the -electron conjugation in 2D planar graphitic carbon nitride: Efficient charge separation for overall water splitting, *Journal of Materials Chemistry A. 2019, 7, 3757-3771.*

(8) Z. H. Li, S. Y. Zhou, Q. Yang, Z. G. Zhang, X. M. Fang, Insight Into the Enhanced Hydrogen Evolution Activity of 2,4-Diaminopyrimidine-Doped Graphitic Carbon Nitride Photocatalysts, *The Journal of Physical Chemistry C. 2019, 123, 2228-2237.*

(9) Y. Wei, J. Z. Zhang, Q. Zheng, J. Miao, P. J. Alvarez, M. C. Long, Quantification of photocatalytically-generated hydrogen peroxide in the presence of organic electron donors: interference and reliability considerations, *Chemosphere. 2021, 279, 130556.*

(10) Y. Shiraishi, S. Kanazawa, Y. Kofuji, H. Sakamoto, S. Ichikawa, S. Tanaka, T. Hirai, Sunlight-Driven Hydrogen Peroxide Production from Water and Molecular Oxygen by Metal-Free Photocatalysts, *Angewandte Chemie International Edition. 2014, 53, 13454-13459.*

(11) Y. Kofuji, S. Ohkita, Y. Shiraishi, H. Sakamoto, S. Tanaka, S. Ichikawa, T. Hirai, Graphitic Carbon Nitride Doped with Biphenyl Diimide: Efficient Photocatalyst for Hydrogen Peroxide Production from Water and Molecular Oxygen by Sunlight, *ACS Catalysis. 2016, 6, 7021-7029.*

(12) Y. Kofuji, Y. Isobe, Y. Shiraishi, H. Sakamoto, S. Tanaka, S. Ichikawa, T. Hirai, *Journal of the American Chemical Society. 2016, 138, 10019-10025.*

(13) Y. Kofuji, S. Ohkita, Y. Shiraishi, H. Sakamoto, S. Ichikawa, S. Tanaka, T. Hirai, Carbon nitride–aromatic diimide-graphene nanohybrids: metalFree photocatalysts for solar-to-hydrogen peroxide energy conversion with 0.2% efficiency, *ACS Sustainable Chemistry & Engineering. 2017, 5, 6478-6485.*

(14) Y. Kofuji, Y. Isobe, Y. Shiraishi, H. Sakamoto, S. Ichikawa, S. Tanaka, T. Hirai, Hydrogen Peroxide production on carbon nitride-boron nitride reduced graphene oxide hybrid photocatalyst under visible light, *ChemCatChem, 2018, 10, 2070-2077.*

(15) L. Chen, L. Wang, Y. Y. Wan, Y. Zhang, Z. M. Qi, X. J. Wu, H. X. Xu, Acetylene and diacetylene functionalized covalent triazine frameworks as metal-free photocatalysts for hydrogen peroxide production: A new two-electron mater oxidation pathway, *Advanced Materials. 2019, 32, 1904433.*

(16) Y. Shiraishi, T. Takii, T. Hagi, S. Mori, Y. Kofuji, Y. Kitagawa, S. Tanaka, S. Ichikawa, T. Hirai, Resorcinol-formaldehyde resins as metal-free semiconductor photocatalysts for solar-tohydrogen peroxide energy conversion, *Nature materials. 2019, 18, 985-993.*

(17) Q. Tian, L. Y. Jing, S. Ye, J. X. Liu, R. T. Chen, C. A. H. Price, F. T. Fan, J. Liu, Nanospatial Charge Modulation of Monodispersed Polymeric Microsphere Photocatalysts for Exceptional Hydrogen Peroxide Production, *Small. 2021, 2103224.*

(18) Y. Shiraishi, T. Hagi, M. Matsumoto, S. Tanaka, S. Ichikawa, T. Hirai, Solar-to-hydrogen peroxide energy conversion onresorcinol-formaldehyde resin photocatalystsprepared by acid-catalysed polycondensation, *Communications Chemistry. 2020, 3, 169.*

(19) Y. Shiraishi, M. Matsumoto, S. Ichikawa, S. Tanaka, T. Hirai, Polythiophene-Doped Resorcinol-Formaldehyde Resin Photocatalysts for Solar-to-Hydrogen Peroxide Energy Conversion, *Journal of the American Chemical Society. 2021, 143, 12590-12599.*

(20) Y. H. Song, R. Y. Ma, L. Hao, X. M. Yang, C. Wang, Q. H. Wu, Z. Wang, Application of covalent organic framework as the adsorbent for solid-phase extraction of trace levels of pesticide residues prior to high-performance liquid chromatography-ultraviolet detection, *Journal of Chromatography A. 2018, 1572, 20-26.*

(21) Y. H. Pang, Y. Y. Huang, X. F. Shen, Y. Y. Wang, Electro-enhanced solid-phase microextraction with covalent organic framework modified stainless steel fiber for efficient adsorption of bisphenol A, *Analytica Chimica Acta. 2021, 1142, 99-107.*

(22) S. Chandra, S. Kandambeth, B. P. Biswal, B. Lukose, S. M. Kunjir, M. Chaudhary, R. Babarao, T. Heine, R. Banerjee, Chemically stable multilayered covalent organic nanosheets from covalent organic frameworks via mechanical delamination, *Journal of the American Chemical Society. 2013, 135, 17853-17861.*

(23) B. P. Biswal, S. Chandra, S. Kandambeth, B. Lukose, T. Heine, R. Banerjee, Mechanochemical Synthesis of Chemically Stable Isoreticular Covalent Organic FrameworksFacile room-temperature solution-phase synthesis of spherical covalent organic framework for high-resolution chromatographic separation, *Journal of the American Chemical Society. 2013, 135, 5328-5331.*
